# Loss of PHD3 allows tumours to overcome hypoxic growth inhibition and sustain proliferation through EGFR

**DOI:** 10.1038/ncomms6582

**Published:** 2014-11-25

**Authors:** Anne-Theres Henze, Boyan K. Garvalov, Sascha Seidel, Angel M. Cuesta, Mathias Ritter, Alina Filatova, Franziska Foss, Higinio Dopeso, Clara L. Essmann, Patrick H. Maxwell, Guido Reifenberger, Peter Carmeliet, Amparo Acker-Palmer, Till Acker

**Affiliations:** 1 Institute of Neuropathology, University of Giessen, 35392 Giessen, Germany; 2 Institute of Cell Biology and Neuroscience, Buchmann Institute for Molecular Life Sciences (BMLS), University of Frankfurt, 60438 Frankfurt, Germany; 3 Focus Program Translational Neurosciences (FTN), University of Mainz, 55131 Mainz, Germany; 4 Cambridge Institute for Medical Research, University of Cambridge, Cambridge CB2 0XY, UK; 5 Institute of Neuropathology, Heinrich Heine University Düsseldorf, 40225 Düsseldorf, German Cancer Consortium (DKTK), German Cancer Research Center (DKFZ), 69120 Heidelberg, Germany; 6 Vesalius Research Center (VRC), Angiogenesis and Neurovascular Link Laboratory, University of Leuven, Leuven B-3000, Belgium

## Abstract

Solid tumours are exposed to microenvironmental factors such as hypoxia that normally inhibit cell growth. However, tumour cells are capable of counteracting these signals through mechanisms that are largely unknown. Here we show that the prolyl hydroxylase PHD3 restrains tumour growth in response to microenvironmental cues through the control of EGFR. PHD3 silencing in human gliomas or genetic deletion in a murine high-grade astrocytoma model markedly promotes tumour growth and the ability of tumours to continue growing under unfavourable conditions. The growth-suppressive function of PHD3 is independent of the established PHD3 targets HIF and NF-κB and its hydroxylase activity. Instead, loss of PHD3 results in hyperphosphorylation of epidermal growth factor receptor (EGFR). Importantly, epigenetic/genetic silencing of PHD3 preferentially occurs in gliomas without EGFR amplification. Our findings reveal that PHD3 inactivation provides an alternative route of EGFR activation through which tumour cells sustain proliferative signalling even under conditions of limited oxygen availability.

## Abstract (web summary)

Little is known on how solid tumours overcome growth inhibitory signals within its hypoxic microenvironment. Here Henze *et al.* show that oxygen sensor PHD3 is frequently lost in gliomas, and that this loss hyperactivates EGFR signaling to sustain tumour cell proliferation and survival in hypoxia.

## Graphical abstract

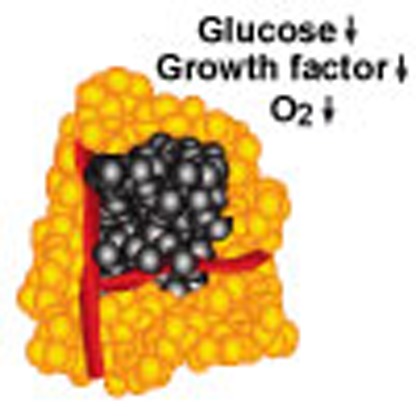

Growing tumours frequently exist within a hypoxic tumour microenvironment because of insufficient blood supply. Hypoxia initiates a wide array of adaptive cellular responses that ultimately promote a more aggressive tumour phenotype. In nonmalignant tissues hypoxia induces a process termed oxygen conformance that is associated with decreased proliferation and enables cell survival under conditions when energy becomes scarce^1^. Little is known, however, about how tumour cells are able to overcome and counteract the growth inhibitory effects of hypoxia to sustain their aberrant growth. The cellular response to hypoxia is primarily mediated by the hypoxia-inducible factors (HIFs)^2^. HIF abundance is tightly regulated by the prolyl hydroxylase domain proteins 1–3 (PHDs; also called EglN)^3^,^4^, which hydroxylate prolyl residues within the oxygen-dependent degradation domain of HIFs^5^. In addition, PHD substrates other than HIF, and PHD functions independent of its enzymatic activity, are being increasingly identified^6^,^7^,^8^,^9^,^10^,^11^. Mechanistic insight into the function of PHDs in tumourigenesis remains limited. Both pro- and antitumourigenic functions have been attributed to PHD1 (refs 12, 13) and PHD2 (refs 6, 14, 15), while recent work suggests a role for PHD3 in suppressing the growth of diverse tumour types^11^,^16^,^17^,^18^. Apart from hypoxia, which is a strong stimulus for PHD3 expression^19^, PHD3 abundance is regulated by other stress-related mechanisms such as growth factor deprivation^20^. These features may allow PHD3 to act as a key sensor of stress signals within the tumour microenvironment. We therefore investigated by which mechanisms inactivation of PHD3 may enable tumours to sustain their growth and overcome growth inhibitory signals within the hypoxic microenvironment.

## Results

### PHD3 is silenced in glioma progression

**Figure 1:**
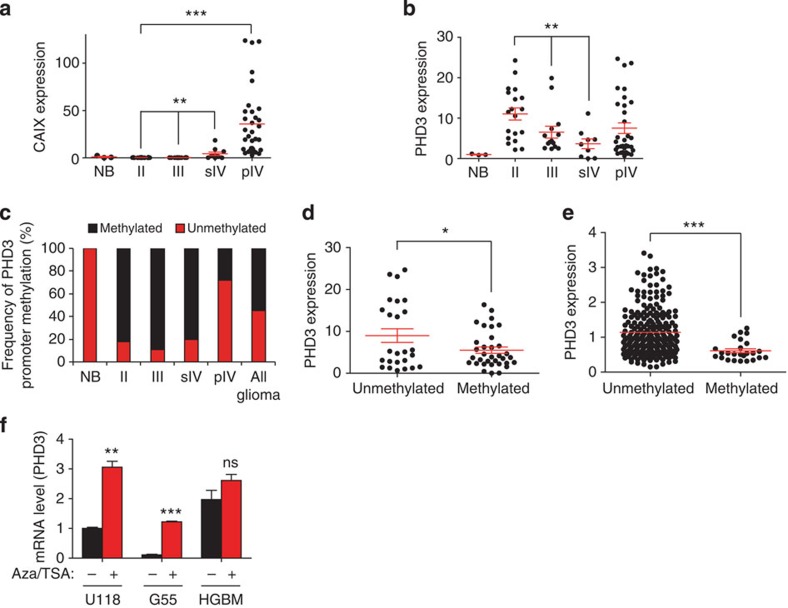
PHD3 is silenced in glioma progression. (**a**,**b**) CAIX expression is enhanced in glioma progression, whereas PHD3 expression is attenuated. qPCR analysis of CAIX (**a**) and PHD3 (**b**) gene expression in normal adult brain (NB), diffuse astrocytomas (WHO grade II), anaplastic astrocytomas (WHO grade III), (s)econdary and (p)rimary glioblastomas (WHO grade IV; *n*=76). (**c**) PHD3 promoter hypermethylation is an early event in glioma progression. Frequency of PHD3 promoter hypermethylation in NB and gliomas of different grades as determined by methylation-specific PCR. (**d**,**e**) PHD3 promoter hypermethylation decreases PHD3 expression. Comparison of PHD3 mRNA levels in gliomas with or without PHD3 promoter methylation as determined in **b**,**c**, respectively, within our glioma cohort (**d**). Comparison of PHD3 mRNA expression in glioblastomas with or without PHD3 promoter methylation from TCGA cohort (*n*=251, tumours for which methylation data are available) (**e**). (**f**) qPCR analysis of PHD3 expression in G55, U118 and HGBM tumour cells with hypermethylated and nonmethylated PHD3 promoter, respectively, ±treatment with the demethylating agent 5-azacytidine (20 μM) for 72 h and Trichostatin A (1 μM) for the last 24 h (*n*=3). All values are means+s.e.m., *P<0.05; **P<0.01; ***P<0.001.

We first examined PHD3 mRNA expression levels during glioma progression in a panel of 76 WHO (World Health Organization) grade II–IV glioma patients. Despite a strong induction of the hypoxic marker CAIX in primary and secondary glioblastomas, mRNA levels of PHD3, which can be highly upregulated by hypoxia^19^, remained unchanged or were even significantly lower, respectively, compared with low-grade gliomas (WHO grade II; Fig. 1a,b). These results suggested that PHD3 expression levels are attenuated in glioma progression and, importantly, are kept low even though tumours activate the hypoxic response. We therefore examined whether PHD3 was genetically or epigenetically inactivated in gliomas. Copy number analysis revealed that the PHD3 genomic region was within a large region of deletion in over 20% of all gliomas from different cohorts^21^,^22^ (Supplementary Fig. 1a–c). Furthermore, PHD3 genetic loss was associated with downregulation of PHD3 expression (Supplementary Fig. 1d), suggesting that single-copy loss of PHD3 may contribute in part to clonal selection of cells carrying this broad deletion. We next assessed whether PHD3 may also be epigenetically silenced in gliomas by promoter hypermethylation, as has been recently reported in multiple myeloma^16^. Methylation-specific PCR (Supplementary Fig. 1e) revealed that PHD3 CpG sites were methylated in more than 80% of all patients with low-grade and anaplastic astrocytomas as well as secondary glioblastomas (Fig. 1c) and to a lower degree in primary glioblastoma patients. Notably and in line with an attenuation of PHD3 expression by promoter methylation, gliomas with increased PHD3 CpG methylation exhibited significantly lower PHD3 levels (Fig. 1d). These findings were corroborated with the TCGA glioblastoma cohort (Fig. 1e, Supplementary Fig. 1f). Importantly, treatment with the DNA methyltransferase inhibitor 5-Azacytidine (5-AzaC) and the histone deacetylase inhibitor trichostatin A (TSA) significantly upregulated PHD3 expression in glioma cell lines with a methylated promoter (Fig. 1f, Supplementary Fig. 1g), supporting the role of promoter methylation in the control of PHD3 expression. Taken together, these results show that PHD3 expression is frequently downregulated by both genetic deletion and promoter hypermethylation.

### Loss of PHD3 increases tumour growth

**Figure 2:**
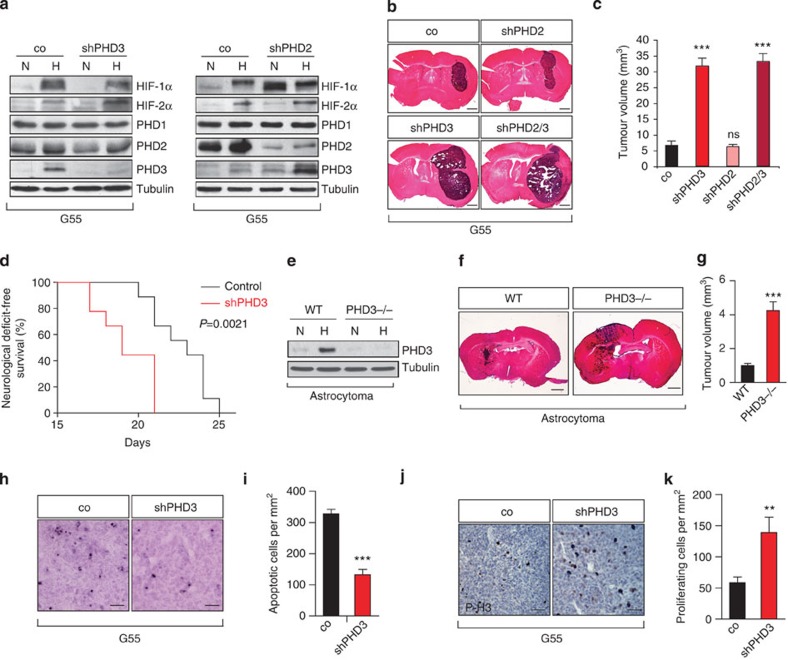
Loss of PHD3 increases tumour growth. (**a**) Immunoblot of G55 glioblastoma cells stably transduced with control (co), PHD2 or PHD3 shRNA following exposure to 21% (*N*) or 1% O_2_ (*H*) for 24 h. (**b**,**c**) PHD3, but not PHD2, loss promotes intracranial glioma growth. Tumour xenografts of polyclonal G55 pools expressing control, PHD2, PHD3 or PHD2/PHD3 shRNA were stained with haematoxylin and eosin (HE; *n*=8). (**d**) Kaplan–Meier survival curves of nude mice intracranially injected with polyclonal G55 pools expressing control or PHD3 shRNA. (**e**) Immunoblot of wild-type (WT) and PHD3^−/−^ astrocytoma cells following exposure to 21% (*N*) or 1% O_2_ (*H*) for 24 h. (**f**,**g**) Genetic inactivation of PHD3 increases mouse astrocytoma growth (*n*=9–10). (**h**,**i**) PHD3 silencing reduces tumour cell apoptosis in xenografts of polyclonal G55 pools (*n*=7–8) assessed by quantifying the number of TUNEL-positive cells per tumour area. (**j**,**k**) Silencing of PHD3 increases cell proliferation in xenografts of polyclonal G55 pools (*n*=8) quantified as the number of phospho-histone 3-positive cells per tumour area. Western blots images (**a**,**e**) have been cropped for presentation. Full size images are presented in Supplementary Fig. 10. All values are means+s.e.m., *P<0.05; **P<0.01; ***P<0.001. Scale bars, 1 mm (**b**,**f**), 50 μm (**h**,**j**).

To gain insight into the role of PHD3 downregulation in tumour growth, we generated lentiviral knockdown G55 glioblastoma cells for PHD3 (Fig. 2a, Supplementary Fig. 2a). Lentiviral PHD3 silencing elevated HIF-2α levels during acute hypoxia (Fig. 2a) and allowed HIF-1α and HIF-2α accumulation during chronic hypoxia (Supplementary Fig. 2b), confirming the functionality of the knockdown. PHD3-deficient G55 cells revealed a strikingly accelerated tumour growth and decreased survival in an intracranial xenograft model (Fig. 2b–d). Within 2 weeks, shPHD3 tumours grew to a fivefold larger size than control tumours. This effect was specific for the PHD3 isoform, as PHD2 knockdown did not alter tumour growth (Fig. 2a–c). We corroborated our results in the primary glioblastoma cell line GBM046x, isolated from a patient biopsy, in which PHD3 silencing significantly promoted intracranial tumour growth (Supplementary Fig. 3a–c). To confirm our findings of a tumour growth-suppressive role of PHD3 in a PHD3 null background, we genetically inactivated PHD3 in a mouse glioma model. We generated murine high-grade astrocytomas lacking PHD3 by Cre-recombination in immortalized and transformed astrocytes^23^ isolated from PHD3^flox/flox^ mice (Fig. 2e, Supplementary Fig. 13). Intracranial injection of PHD3^−/−^ astrocytomas yielded tumours with a highly increased growth and over fourfold larger volume than control tumours (Fig. 2f,g).

### PHD3 regulates tumour cell apoptosis and proliferation

Next we sought to understand how PHD3 disruption promotes tumour growth. The acquisition of a vascular supply is rate-limiting for tumour growth and is centrally regulated by the PHD/HIF system. Therefore, we investigated whether PHD3 deficiency affects tumour angiogenesis. Analysis of tumour blood vessels did not reveal a significant difference in vessel density or vessel morphology between control and PHD3-deficient tumours (Supplementary Fig. 4) indicating that the tumour growth-promoting effects of PHD3 loss are independent of the regulation of tumour vascularization. Consistently, vascular endothelial growth factor (VEGF) mRNA levels were not increased following PHD3 silencing (Supplementary Fig. 2a). Instead, the increased growth of PHD3-silenced G55 tumours could be attributed to a combination of decreased tumour cell apoptosis as assessed with TdT-mediated dUTP nick end labeling (TUNEL) staining (Fig. 2h,i) and increased cell proliferation as determined by the proliferation marker phospho-histone H3 (Fig. 2j,k). These results were corroborated in tumours originating from the PHD3-silenced primary glioblastoma cell line GBM046x (Supplementary Fig. 5a–d) and from the PHD3-/- astrocytomas (Supplementary Fig. 5e–h). These data suggest that PHD3 loss promotes resistance to apoptosis induction and increases cell proliferation.

**Figure 3:**
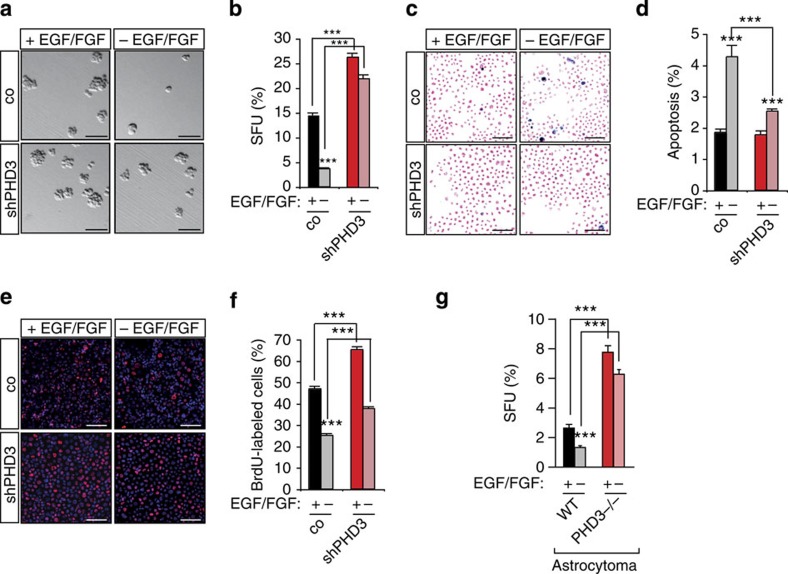
Loss of PHD3 promotes tumour cell survival and proliferation. (**a**,**b**) PHD3 silencing increases clonal cell growth in a 3D spheroid culture system. G55 cells expressing control or PHD3 shRNA were cultured as spheroids in B27-supplemented serum-free medium±EGF/FGF and the number of spheroids was quantified after 3 days (*n*=6). (**c**,**d**) PHD3 loss protects against cell death induction following growth factor withdrawal. Apoptosis was assessed by quantifying the number of TUNEL-positive cells after 48 h of incubation in B27-supplemented serum-free medium±EGF/FGF (*n*=15). (**e**,**f**) PHD3 loss increases cell proliferation. Cell proliferation was assessed by quantifying the number of 5-bromodeoxyuridine-positive cells after 48 h of incubation in B27-supplemented serum-free medium±EGF/FGF (*n*=15). (**g**) PHD3 disruption by genetic inactivation confers a growth advantage in additional glioma cell systems. Clonal cell growth was quantified as in **b** in the presence or absence of EGF/FGF in PHD3^−/−^ astrocytoma cells (*n*=6). All values are means+s.e.m., **P<0.01; ***P<0.001. Scale bars, 50 μm.

We next set up an *in vitro* assay to mechanistically dissect the PHD3-dependent control of tumour cell growth. We used a three-dimensional (3D) tumour spheroid culture system under defined serum-independent conditions that more closely replicates the growth characteristics of tumours *in vivo* and sensitively responds to changes in growth factor concentration. The number of spheres as a parameter of clonal cell growth was significantly increased by PHD3, but not by PHD2 inactivation following EGF/FGF stimulation (Fig. 3a,b, Supplementary Fig. 6a). The capacity to sustain proliferative signalling is one of the hallmarks of malignancy^24^ and can be tested *in vitro* by the ability of tumour cells to grow under growth factor-deprived conditions. While sphere formation was drastically reduced upon growth factor withdrawal in control tumour cells, PHD3 inactivation allowed tumour cells to continue growing in the absence of exogenous growth factors (Fig. 3a,b). Correspondingly and in line with the *in vivo* results, PHD3 loss almost completely protected cells against cell death induction under starving conditions (Fig. 3c,d) and enhanced cell proliferation both in the presence and in the absence of growth factors (Fig. 3e,f). The effect of PHD3 inactivation on cell growth was confirmed in PHD3^−/−^ astrocytomas (Fig. 3g), two additional glioblastoma cell lines (G141 and HGBM), as well as the primary line GBM046x (Supplementary Fig. 6b–d). Taken together, these data establish that PHD3 is a critical regulator of cell growth and survival and that PHD3 deficiency in gliomas confers a pronounced growth advantage *in vitro* and *in vivo*.

### PHD3 mediates growth inhibition

**Figure 4:**
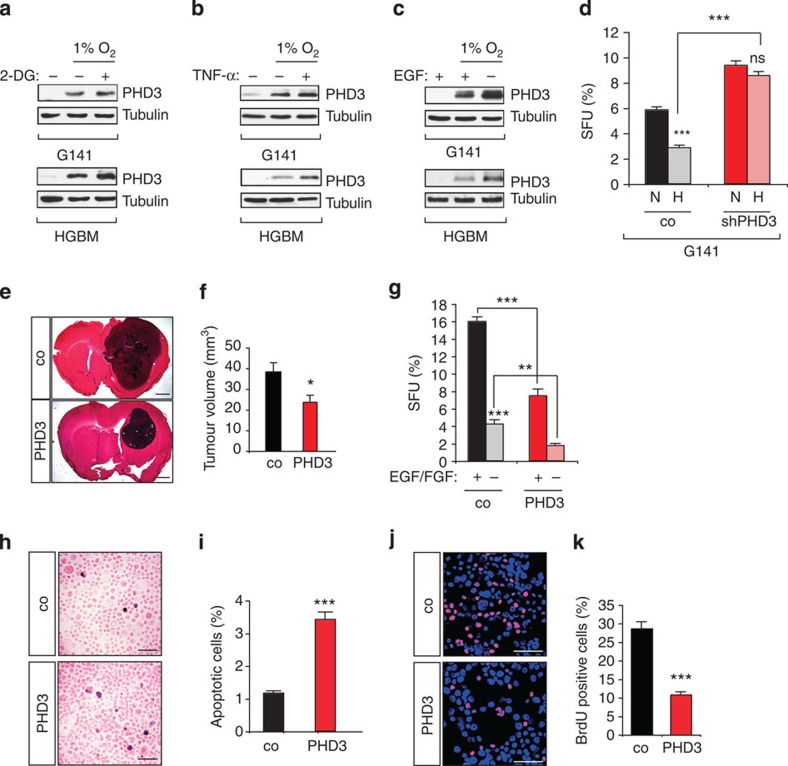
PHD3 is a mediator of hypoxic growth inhibition. (**a**–**c**) Growth inhibitory signals induce PHD3 expression. Immunoblot of G141 and HGBM tumour cells following exposure to 21% (N) or 1% O_2_ (H) for 24 h, in combination with ±hypoglycaemia (treatment with 6 mM 2-deoxyglucose) (**a**), incubation with TNFα (10 ng ml^−1^) (**b**) or EGF (20 ng ml^−1^) (**c**). (**d**) PHD3 loss protects against hypoxic growth inhibition. Clonal spheroid growth (*n*=6) of G141 tumour cells expressing control or PHD3 shRNA was quantified following exposure to 21% (N) or 1% O_2_ (H) for 3 days. (**e**,**f**) PHD3 inhibits intracranial glioma growth. Tumour xenografts of polyclonal G55 pools expressing PHD3 or GFP control were stained with HE (**e**) and the tumour volume was quantified (**f**) (*n*=9–10). (**g**) PHD3 decreases clonal cell growth in a 3D spheroid culture system. G55 cells expressing PHD3 or GFP control were cultured as spheroids in B27-supplemented serum-free medium±EGF/FGF and the number of spheroids was quantified after 3 days (*n*=6). (**h**,**i**) PHD3 expression enhances apoptosis. Cell apoptosis was assessed in G55 pools expressing PHD3 or GFP control by quantifying the number of TUNEL-positive cells after 48 h of incubation in B27-supplemented serum-free medium+EGF/FGF (*n*=18). (**j**,**k**) PHD3 inhibits proliferation. Cell proliferation was assessed in G55 pools expressing PHD3 or GFP control by quantifying the number of BrdU-positive cells after 48 h of incubation in B27-supplemented serum-free medium+EGF/FGF (*n*=15). Western blot images (**a**–**c**) have been cropped for presentation. Full size images are presented in Supplementary Fig. 10. All values are means+s.e.m., *P<0.05; **P<0.01; ***P<0.001. Scale bars, 1 mm (**e**), 50 μm (**h**,**j**).

Given the importance of PHD3 in growth control, we wanted to test the hypothesis that changes in PHD3 levels may allow cells to direct the cell growth response. Except for hypoxia, little is known about additional stimuli that regulate PHD3 expression. We therefore first determined whether PHD3 levels may be flexibly altered by growth-stimulating and -inhibiting conditions. Notably, hypoxic treatment, induction of hypoglycaemia with 2-deoxyglucose or incubation with the cytokine tumour-necrosis factor (TNF)-α (Fig. 4a,b, Supplementary Fig. 7a,b) strikingly increased PHD3 levels, whereas growth stimulation with EGF reduced PHD3 levels (Fig. 4c, Supplementary Fig. 7b), indicating that growth inhibitory conditions stimulate PHD3 expression. We next addressed whether increased PHD3 levels could relay the growth inhibitory signals. While hypoxia strikingly reduced total cell number and sphere-forming capacity in the glioma cell line G141, which robustly induces PHD3 under hypoxia, PHD3 loss allowed for a continued sphere formation and cell accumulation under hypoxia (Fig. 4d, Supplementary Fig. 7a–d). To functionally corroborate this role of PHD3 in tumour growth suppression we used G55 cells with increased PHD3 expression levels, and control G55 cells expressing green fluorescent protein (GFP). Increased PHD3 expression significantly reduced intracranial tumour growth (Fig. 4e,f), confirming the growth-suppressive function of PHD3. Similarly, PHD3 decreased sphere formation even in the presence of growth factors (Fig. 4g), concomitantly with an increase in cell death and a decrease in proliferation (Fig. 4h,k). Collectively, these results demonstrate that tumour growth responses are highly sensitive to PHD3 expression levels, indicating that PHD3 is a key regulator of tumour growth control in response to microenvironmental cues.

### PHD3-mediated growth control is hydroxylase-independent

**Figure 5:**
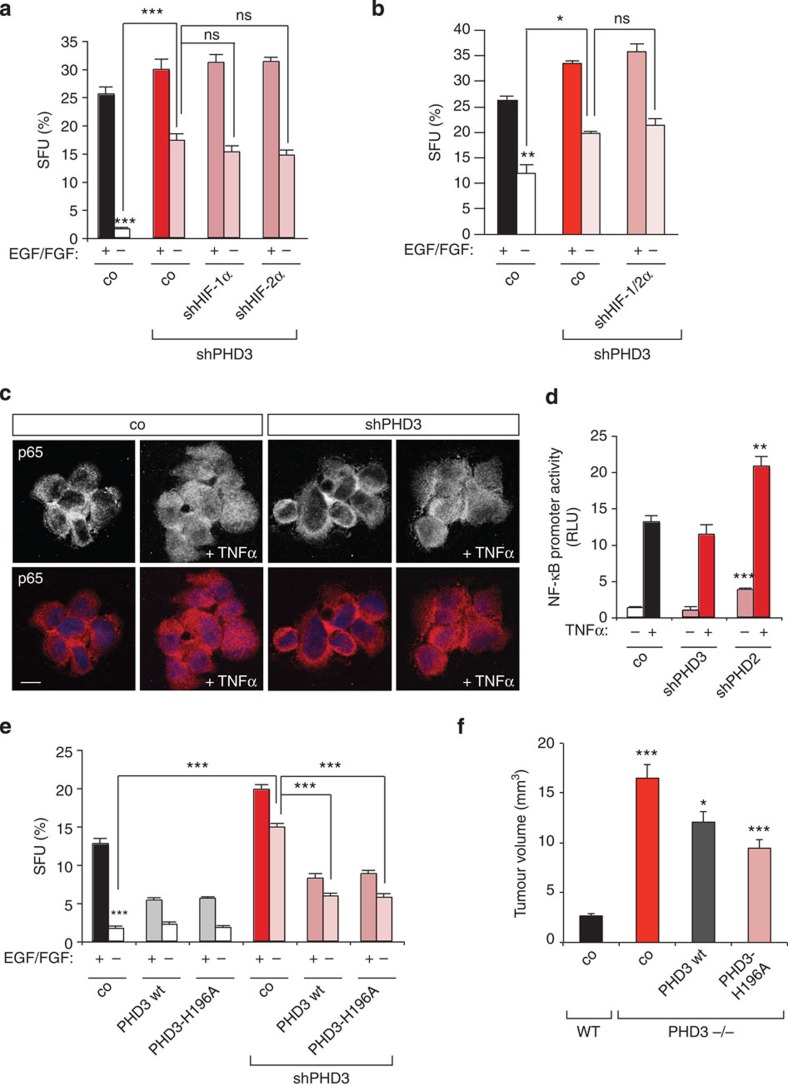
PHD3 function in tumour growth is hydroxylase-independent. (**a**,**b**) The growth-promoting effect of PHD3 loss is HIF-1/2α-independent. G55 cells expressing control or PHD3 shRNA in combination with control, HIF-1α or HIF-2α (**a**) or double HIF-1α/2α (**b**) shRNA were cultured as spheroids in B27-supplemented serum-free medium±EGF/FGF and the number of spheroids was quantified after 3–5 days (*n*=6). (**c**,**d**) NF-κB signalling is not altered following PHD3 loss. G55 cells expressing control or PHD3 shRNA were cultured as spheroids±treatment with TNF-α (10 ng ml^−1^, 30 min). Immunofluorescence with anti-p65 antibodies shows that basal and TNF-α-stimulated translocation of p65 to the nucleus is not affected by PHD3 loss (**c**). PHD3 loss does not activate NF-κB signalling. G55 cells expressing control, PHD3 or PHD2 (positive control) shRNA were transfected with an NF-κB luciferase reporter construct±treatment with TNF-α (10 ng ml^−1^, 6 h) and EGF (20 ng ml^−1^, 6 h; *n*=3) (**d**). (**e**) The hydroxylase function of PHD3 is not required for the regulation of tumour cell growth. G55 cells expressing PHD3 or control shRNA were transfected with either wild-type PHD3, the hydroxylase mutant PHD3-H196A or empty vector control, cultured as spheroids in B27-supplemented serum-free medium±EGF/FGF and the number of spheroids was quantified after 3 days (*n*=6). (**f**) PHD3 wild-type and hydroxylase mutant decrease the PHD3-loss-mediated increase in intracranial tumour growth (*n*=9–10). All values are means+s.e.m., *P<0.05; **P<0.01; ***P<0.001. Scale bars, 10 μm.

We next addressed the mechanisms by which PHD3 disruption sustains proliferative signalling. Importantly, we found that the growth-promoting effects of PHD3 inactivation were independent of HIF or NF-κB activation, two major downstream mediators of PHD3 function^3^,^4^,^7^,^11^,^25^. Silencing of HIF1α and/or HIF2α did not abolish the growth advantage conferred by PHD3 loss (Fig. 5a,b, Supplementary Fig. 8a,b). While silencing of PHD2 markedly increased nuclear factor kappa-light-chain-enhancer of activated B cells (NF-κB) transcriptional activity, NF-κB activation was not altered following PHD3 loss (Fig. 5c,d), which is supported by previous findings^7^ and indicates that NF-κB is not involved in mediating the growth-promoting functions of PHD3 disruption. Furthermore, the hydroxylase activity of PHD3 was also not required for the control of tumour growth, as expression of both wild-type PHD3 and PHD3 mutants with substitutions of critical residues in the catalytic hydroxylase domain, H196A and H135A/D137A^3^,^9^,^20^ similarly suppressed the growth of PHD3-deficient and control tumour cells (Fig. 5e, Supplementary Fig. 8c,d). Moreover, the increased *in vivo* growth of PHD3-deficient tumours could be partially rescued by both the wild-type and the hydroxylase-deficient PHD3 mutant (Fig. 5f).

### PHD3 loss sustains proliferative signalling through EGFR

**Figure 6:**
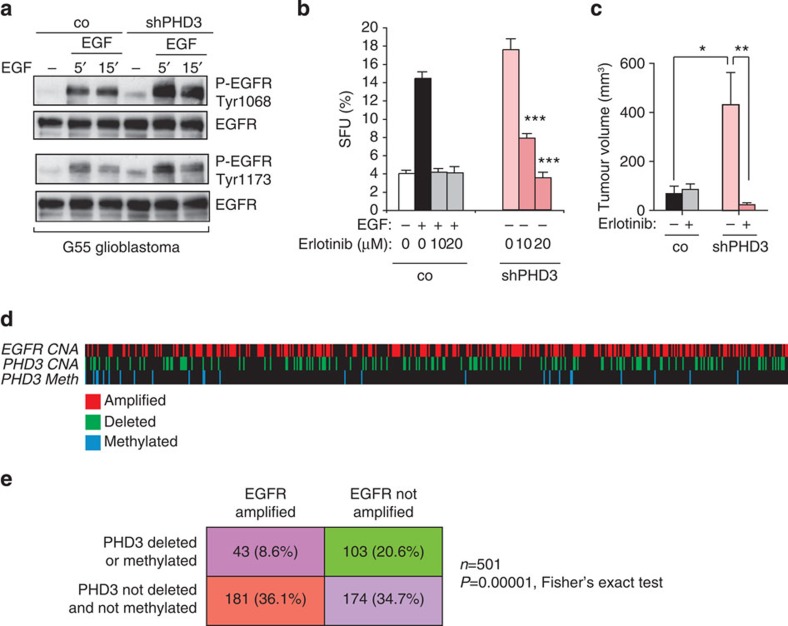
PHD3 loss sustains proliferative signalling through EGFR. (**a**) EGFR phosphorylation is enhanced following PHD3 loss. Immunoblot with two different phospho-specific antibodies against EGFR of G55 cells expressing PHD3 or scrambled control nonstimulated or stimulated with EGF (20 ng ml^−1^) for the indicated times. (**b**,**c**) EGFR activity is required for the growth-promoting effect of PHD3 loss. G55 cells expressing control or PHD3 shRNA were cultured as spheroids in B27-supplemented serum-free medium±EGF (20 ng ml^−1^). Where indicated, cells were pretreated with the EGFR inhibitor erlotinib (10, 20 μM) for 2 h before stimulation. The number of spheroids was quantified after 3 days (*n*=6; **b**). Nude mice transplanted with G55 cells expressing control or PHD3 shRNA were treated with vehicle or erlotinib and tumour growth was quantified (*n*=13–18; **c**). (**d**,**e**) PHD3 loss through deletion or promoter methylation occurs preferentially in glioblastomas without EGFR amplification. Analysis of CNA for EGFR and PHD3, as well as PHD3 promoter methylation (Meth) in glioblastomas from the TCGA cohort (*n*=501, tumours for which CNA data are available). High-level EGFR amplification is shown in *red*, PHD3 deletion in *green* and PHD3 promoter methylation in *blue* (**d**). PHD3 deletion or promoter methylation are significantly more common in tumours without EGFR amplification (**e**). Western blot images (**a**) have been cropped for presentation. Full size images are presented in Supplementary Fig. 11. All values are means+s.e.m., *P<0.05; **P<0.01; ***P<0.001.

The sustained proliferation and the growth signal autonomy of PHD3-deficient tumour cells indicated that PHD3 inactivation may affect growth factor signalling pathways. The dysregulation of receptor tyrosine kinase (RTK) pathways is central to the process of malignant transformation. We therefore performed an unbiased screen under nonstimulated conditions to detect basal changes in phosphorylation of RTKs using an antibody-based Phospho-RTK array (Supplementary Fig. 9a). Interestingly, tyrosine phosphorylation of epidermal growth factor receptor (EGFR) was selectively increased following PHD3 loss under basal conditions (Supplementary Fig. 9a) as well as after stimulation with EGF (Supplementary Fig. 9b). In agreement with a specific effect on EGFR phosphorylation, PHD3 loss did not result in an overall increase in tyrosine phosphorylation (Supplementary Fig. 9b). On the contrary, phosphorylation of other RTKs including PDGFRα, VEGFR1 and EphB2 was even reduced (Supplementary Fig. 9a). The apparent increased phosphorylation of Axl was not confirmed when using specific antibodies for this receptor (data not shown). We next confirmed that PHD3 alters EGFR phosphorylation using two different phospho-specific antibodies for EGFR Tyr 1068 and Tyr 1173, which serve as docking sites for the adaptors Grb2 and Shc and are crucial for the biological activity of EGFR^26^. Notably, PHD3-silenced tumour cells exhibited a striking and prolonged increase in EGFR phosphorylation under basal conditions as well as after EGF stimulation for 5 and 15 min (Fig. 6a). Since EGFR signalling has a central role in conveying cell growth autonomy and malignant transformation in gliomas^27^, we next investigated whether the growth-promoting effects of PHD3 inactivation are functionally dependent on the increased EGFR activity. Treatment with the RTK inhibitor erlotinib abolished the increased EGFR phosphorylation and abrogated the growth-promoting effect of PHD3 loss on sphere formation, illustrating the requirement of EGFR activity for the growth-promoting function of PHD3 loss (Fig. 6b and Supplementary Fig. 9c). We next assessed whether the increased EGFR activity is also functionally required for the tumour growth-promoting effect of PHD3 inactivation *in vivo*. Control and PHD3-deficient G55 glioblastoma cells were injected in nude mice, which were treated with vehicle or erlotinib. Notably, the growth advantage conferred by PHD3 loss was fully abolished by erlotinib treatment (Fig. 6c), confirming that the *in vivo* growth-promoting function of PHD3 is dependent on EGFR. Taken together, these data demonstrate that the growth advantage conferred by PHD3 loss is because of hyperactivation of EGFR signalling, suggesting a molecular crosstalk between PHD3 and EGFR signalling.

### PHD3 is lost in glioblastomas without EGFR amplification

As our findings show that the tumor growth-promoting effect of PHD3 silencing is exerted through activation of EGFR, we next sought to determine how PHD3 inactivation relates to other mechanisms that enhance EGFR signalling, for example, genetic EGFR amplification, which is a characteristic feature of primary glioblastoma^27^. We found that, while a high level of EGFR amplification was observed in around half of the glioblastoma patients from the TCGA cohort, PHD3 suppression through deletion and/or promoter methylation was much more common in the tumors without EGFR amplification: over 70% of the patients with PHD3 deletion and/or promoter methylation did not have an EGFR amplification, while over 80% of the patients with an EGFR amplification did not harbour a PHD3 deletion and/or promoter methylation (Fig. 6d,e). These findings suggest that PHD3 depletion plays a complementary role as an alternative EGFR-activating mechanism and may be particularly relevant for tumours without EGFR amplification.

## Discussion

Carcinogenesis is a multistep process that endows tumour cells with a set of hallmarks including sustained proliferative signalling, refractoriness to growth inhibitory signals and resistance to cell death through a series of genetic, epigenetic and microenvironmental changes^24^. Here we identify PHD3 as a novel regulator of EGFR activity that limits tumour proliferation and survival in response to growth inhibitory cues. Our clinical data suggest that PHD3 loss by genetic/epigenetic alterations could play a complementary role as an alternative EGFR-activating mechanism and may be particularly relevant for tumours without EGFR amplification.

In tumour tissue, hypoxia and nutrient starvation are common phenomena. A fundamental cellular response to hypoxia that enables cell survival is the induction of oxygen conformance through depression of cellular metabolism^28^. As a result, cells decrease their proliferation, enter cell cycle arrest and under some circumstances undergo cell death^29^,^30^,^31^. This dormant state enables cell survival under unfavourable growth conditions. The HIF-1α-dependent increase of the cell cycle inhibitors p21 and p27 (ref. 1), the repression of the hexameric MCM helicase 2–7 (refs 32) or the HIF-independent inhibition of mRNA translation^33^ have been described as important regulators of hypoxia-induced growth suppression that, however, can be counteracted by tumour-specific genetic alterations. Our results identify PHD3 upregulation as an additional mechanism to promote growth inhibition. Increased PHD3 levels lead both to a strong suppression of proliferation and to a pronounced increase in apoptosis. Importantly, we demonstrate that silencing of PHD3 can reverse these effects, resulting in enhanced proliferative capacity and suppressed apoptosis *in vitro* and *in vivo*. Our observations are in line with several reports that recognized PHD3 as a mediator in the cellular survival response, linking PHD3 function to apoptosis induction^20^,^34^,^35^,^36^ and providing evidence for increased resistance to cell death following PHD3 loss^37^,^38^. At the same time however, PHD3 has been implicated as a negative regulator of cell death. Thus, the hypoxia-dependent upregulation of PHD3 together with PHD2 can be part of negative feedback loop that restrains the pro-apoptotic functions of HIF^19^. Notably, the HIF-dependent effects of PHD3 on apoptosis under these conditions are only consistently manifested if PHD2 is co-silenced. Our present work reveals that the silencing of PHD3, which occurs during glioma progression, primarily affects tumor growth by different mechanisms that do not depend on HIF, but centre around the control of EGFR activity, resulting in a net stimulation of cell growth coupled to reduced apoptosis. Thus, our data indicate that PHD3 suppression represents an important step in the transition of tumour cells from a dormant to a proliferative state under hypoxia or other growth-inhibiting conditions.

Two features may allow PHD3 to act as a key regulator of growth control: its robust induction by growth inhibitory signals and its hydroxylase-independent control of tumour growth. PHD3 is most potently upregulated under hypoxic conditions compared with other PHD isoforms^19^,^39^,^40^ and is also induced through growth factor deprivation in neuronal cells^41^. Importantly, we show here that in glioblastoma cells hypoxia cooperates with other growth-inhibiting signals, such as growth factor/nutrient deprivation and treatment with inhibitory cytokines, to robustly upregulate PHD3 expression. Furthermore, the dispensability of PHD3 hydroxylase activity for its HIF-independent tumour-suppressive role in glioblastoma uncouples PHD3 function from the requirement for cofactors (Fe^2+^, ascorbate) and co-substrates (O_2_, 2-oxoglurate). PHD3 is thus able to control cell proliferation over a broad physiological range, since its influence on cellular processes is not constrained by the availability of these rate-limiting factors within the tumour microenvironment. This implies that the control of PHD3 expression levels is critical for tumour growth and progression in particular under hypoxic and nutrient-deprived conditions. Such extrinsic cues dynamically regulate PHD3 levels and could contribute to its downregulation in tumour progression. In addition, genetic and epigenetic mechanisms can also lead to PHD3 depletion to promote tumour growth. Our clinical data show that PHD3 expression is attenuated during glioma progression even though high-grade gliomas are highly hypoxic. The majority of gliomas in our cohort had a methylated PHD3 promoter, and this was associated with a marked decrease in PHD3 expression. The general relevance of this phenomenon is supported by recent reports that have linked PHD3 promoter methylation to impaired expression in B-cell neoplasias, colorectal cancer and various cancer cell lines^16^,^42^,^43^,^44^. In addition, we found genetic deletion of the PHD3 locus in over 20% of gliomas. While the PHD3 locus in these tumours was located within a relatively broad region of deletion, the functional importance of single-copy PHD3 loss was corroborated by the significant reduction in PHD3 expression levels in tumours with genetic deletion of PHD3. It is nevertheless feasible that the loss of additional genes located within this region may also contribute to the selection of cell clones in which it is deleted. Additional functions of PHD3, for example, related to the control of the hypoxic response, are likely to further contribute to the selection of tumour cells with PHD3 loss, as suggested by the fact that epigenetic PHD3 silencing was also observed in low-grade gliomas, where EGFR signalling is not thought to play a major role.

Although the regulation of HIF stability by hydroxylation is the best studied function of the PHD protein family so far, a growing number of studies demonstrate that PHDs also possess important HIF-independent functions. Interestingly, there is particularly abundant evidence of HIF-independent interaction partners and functions for PHD3 (refs 10, 11, 45, 46, 47, 48) including the regulation of the candidate tumour suppressor KIF1Bβ^35^, as well as the stress response protein and transcription factor ATF4 (ref. 8). Our own results show that the growth advantage conferred by silencing of PHD3 was not affected by suppression of HIF proteins, arguing against a role for HIF in this process. Similarly, PHD3 depletion did not affect the activity of the NF-κB pathway, which has also been proposed as a PHD target^7^,^11^,^25^. Several reports indicate the necessity for a functional PHD3 enzyme even for targets different from HIF^8^,^10^,^25^,^35^. At the same time, however, there is also evidence that PHD3 can act in a hydroxylation-independent manner. For example, the tumour-suppressor function of PHD3 in colorectal cancer, which is mediated through an IKKβ/NF-κB-dependent pathway, does not require the enzymatic activity of PHD3 (ref. 11). Our data show that the hydroxylase function of PHD3 is also dispensable for control of tumour growth, as demonstrated by our findings that two PHD3 mutants incapable of hydroxylation phenocopied the growth inhibitory functions of the wild-type protein. Instead, we have discovered that PHD3 loss sustains proliferative signalling in glioblastomas through the upregulation of EGFR phosphorylation. As shown in the accompanying paper^49^, the increased EGFR phosphorylation is a result of a deregulated EGFR endocytosis. The accompanying study^49^ unravels a novel scaffold function for PHD3 that regulates the internalization and signalling of EGFR by controlling the recruitment of the endocytic adaptor machinery to EGFR. Thus, our findings uncover the PHD3-dependent regulation of EGFR as a novel mechanism, extending the range of PHD3 targets to a new signalling pathway with a key role in tumor progression.

In summary, both studies together (see ref. 49) support a model whereby PHD3 levels in tumour cells are flexibly regulated by various growth-promoting and growth inhibitory signals from the tumour microenvironment through the regulation of EGFR signalling. Within this context, genetic deletion and epigenetic silencing would contribute to a downregulation of PHD3 in tumour progression, thereby decreasing the sensitivity to growth inhibitory signals and shifting the overall balance towards growth promotion. Taken together, our results demonstrate that PHD3 downregulation can indeed provide a common mechanism through which tumours overcome and counteract the growth inhibitory effect of hypoxia, allowing aberrant tumour growth even at low oxygen tension.

## Methods

### Cell culture

The glioblastoma cell lines G55TL and G141 were kindly provided by M. Westphal and K. Lamszus (Hamburg, Germany), HGBM by H. Weich (Braunschweig, Germany). Glioblastoma cell lines were cultured in DMEM (Invitrogen) supplemented with 10% fetal bovine serum (PAN Systems). For propagating cells under neurosphere conditions, cell culture dishes, plates or flasks were coated with 10 mg ml^−1^ pHEMA and dried. Neurosphere medium (DMEM-F12, (Gibco, Invitrogen, Carlsbad, CA), 2% B27 Serum-Free Supplement (Invitrogen) was supplemented with 20 ng ml^−1^ bFGF and 20 ng ml^−1^ EGF (PeproTech, Hamburg, Germany). The primary glioblastoma line GBM046x was obtained from a patient undergoing surgery in accordance with a protocol approved by the institutional review board and cultured in neurosphere medium. Following lentiviral transduction cells were cultured with 2–6 μg ml^−1^ blasticidin or 2 μg ml^−1^ puromycin depending on the respective vector system. All cells were maintained at 37 °C in 5% CO_2_. For overexpression, G55TL cells were stably transfected with the pTet-Off regulator plasmid (Clontech) and the pTRE2hyg/pur-PHD2, -PHD3 or -GFP-inducible expression plasmids and selected with 200 μg ml^−1^ hygromycin. For hypoxic treatment cells were grown at 1% O_2_ for the indicated time points in a Hypoxic Workstation (Ruskinn Technology, Pencoed, UK; Coy Lab, Grass Lake, USA).

### Generation of PHD3^flox/flox^ mice

A targeting vector was constructed to introduce loxP sites flanking exons 2 and 3 of murine PHD3, using the parental plasmid pDELBOY that contains an Frt-site-flanked neomycin gene driven by the phosphoglycerate kinase (PGK) promoter, two loxP sites and a cassette containing the herpes simplex virus thymidine kinase gene driven by the PGK promoter. The targeting vector contained (i) the thymidine kinase cassette outside of the homologies to allow for negative selection against random integration events; (ii) a 3.5-kb 5′ homology spanning part of intron 1 up to 316 bp upstream of exon 2; (iii) a 2.6-kb ‘mid’ region comprising the remaining part of intron 1 through the beginning of intron 3 (up to 540 bp downstream of exon 3) flanked by loxP sites; (iv) the Frt-site-flanked neomycin cassette, in reverse orientation with respect to the PHD3 gene fragments; and (v) a 3-kb 3′ homology comprising the remainder of intron 3, exon 4, intron 4 and a major part of exon 5 (up to nt 1,584 of exon 5; Supplementary Fig. 13). The vector was linearized with Not1 before electroporation in B6/129 G4 ES cells. Correctly targeted clones were identified by appropriate Southern blot analysis.

### Generation of PHD3^−/−^ and control murine astrocytomas

High-grade murine astrocytomas were generated from PHD3^flox/flox^ mice, by immortalizing astrocytes with SV-40 large T antigen and transforming them through V-12H-Ras expression, as previously described^23^,^50^. Briefly, primary astrocytes were isolated from P1-2 PHD3^flox/flox^ mice and plated in noncoated polystyrene culture flasks. Astrocytes were purified by shaking the flasks on a rotator at 250 r.p.m. at 37 °C for 2 days to detach all other cells. Cells were washed and the medium was changed every day. After confirmation of purity by staining with anti-glial fibrillary acidic protein (GFAP) antibody, astrocytes were stably co-transfected with SV-40 large T antigen expression construct (pOT-largeT) and the pEYFP-N1 plasmid (Clontech) by electroporation with a nucleofector kit (Amaxa). After selection in medium containing 150 μg ml^−1^ geneticin (Gibco) for 2 weeks, resistant colonies were pooled and transduced with a retrovirus expressing the H-Ras V12 oncogene (pBABE puro H-Ras V12, Addgene). Colonies grown in selection medium containing 2 μg ml^−1^ puromycin (Sigma-Aldrich) were pooled. Expression of SV-40 Large T antigen and H-Ras V12 proteins was confirmed by immunoblot analysis with corresponding antibodies (Calbiochem). The astrocytomas were then transduced with adenovirus expressing Cre recombinase and GFP or GFP alone (Ad-Cre-GFP, Ad-CMV-GFP, Vector Biolabs) to produce PHD3 knockout and wild-type astrocytoma cells, respectively. The transduced GFP-positive cells were purified by fluorescence-activated cell sorting (FACS) using a FACSAria III cell sorter (BD Biosciences).

### Reagents

The following reagents were used: 10 ng ml^−1^ Recombinant Human TNF-α (PeproTech), 6 mM 2-deoxyglucose (Sigma-Aldrich), 1 μM TSA (Sigma-Aldrich), 20 μM 5-AzaC (Sigma-Aldrich), 10–20 μM Erlotinib HCl (Sequoia Research Products).

### Glioma specimens

The investigated glioma specimens were retrieved from the archive of the Department of Neuropathology, Heinrich Heine University, Düsseldorf, Germany, and investigated in an anonymized manner as approved by the institutional review board (study no. 4112). All tumours had been originally classified according to the criteria of the WHO classification of tumours of the nervous system of 2000, which in case of diffuse astrocytic gliomas correspond to those in the current WHO classification^51^. Only tissue specimens with a histologically estimated tumour cell content of 80% or more were used for nucleic acid extraction using ultracentrifugation over caesium chloride. PHD3 gene copy number analysis was performed using array comparative genomic hybridization^22^, and gene expression quantification was performed with quantitative PCR (qPCR) and microarray analysis; qPCR analysis was carried out using cDNA from 33 primary glioblastomas (WHO grade IV), 10 secondary glioblastomas (WHO grade IV), 14 anaplastic astrocytomas (WHO grade III) and 19 diffuse astrocytomas (WHO grade II); microarray expression profiling data were obtained from 41 primary glioblastomas, 9 secondary glioblastomas, 12 anaplastic astrocytomas and 8 diffuse astrocytomas. The methylation status of the PHD3 promoter was determined by methylation-specific PCR in 32 primary glioblastomas, 10 secondary glioblastomas, 9 anaplastic astrocytomas and 11 diffuse astrocytomas. Three different commercially available RNA samples from the adult brain were obtained from Clontech, Stratagene and BioChain, as controls for mRNA expression. As controls for the methylation status of the adult brain, four brain samples from patients who died of noncerebral causes were used.

### Plasmids

pTet-Off was purchased from BD Bioscience Clontech. PHD2, PHD3 and GFP cDNA were subcloned in the pTRE2hyg/pur vector (BD Bioscience Clontech) to generate the pTRE2hyg/pur-PHD3 or –GFP-inducible expression plasmids. The PHD3 expression plasmids HA-EGLN3 wt-pcDNA3 and HA-EGLN3 H196A-pcDNA3 were a kind gift from W. Kaelin (Boston, USA)^20^, the pcDNA-PHD3(H135A/D137A) plasmid was a kind gift from G. Semenza (John Hopkins University, USA)^9^. Empty control vector pcDNA3 was purchased from Invitrogen, pGIPZ lentiviral shRNAmir vectors for HIF-1α, HIF-2α and pGIPZ nonsilencing control were purchased from Open Biosystems. The Block-it Pol II miR RNAi Expression Vector Kit (Invitrogen) was used to construct pcDNA6.2-GW/EmGFP-miR vectors (Invitrogen) expressing the target (PHD3 and PHD2) micro RNAs (short hairpin RNA, shRNAs) according to the manufacturer’s instructions using specific miRNA sequences (see the Supplementary Methods: Primer and shRNA sequences); miRNA/shRNA targeting the Drosophila *SIMA* gene was used as control. The Rapid BP/LR recombination reaction (Block-it Lentiviral Pol II miR RNAi Expression System, Invitrogen) between pDONR 221, pcDNA6.2-GW/EmGFP-miR and pLenti6/V5-DEST was performed to generate the pLenti6/V5-GW/EmGFP-miR expression construct.

### Transfection, virus production and infection

G55TL cells were stably transfected with the pTet-Off regulator plasmid (Clontech) and the pTRE2hyg/pur-PHD3 or -GFP-inducible expression plasmids, selected with 200 μg ml^−1^ hygromycin and screened for low transgene background and high transgene induction by western blot and qPCR analyses.

Lentiviral particles using the pGIPZ vectors were produced according to the manufacturer’s directions using the Trans-Lentiviral shRNA Bulk Packaging System (Open Biosystems). Cells were selected with 2 μg ml^−1^ puromycin to obtain resistant polyclonal cell pools. Lentiviral particles using the Block-it system were produced in HEK293T cells using the ViraPower Lentiviral Expression System (Invitrogen) and Lipofectamine 2000 according to the manufacturer’s instructions or the pCI-VSVG and psPAX2 packaging plasmids and calcium phosphate transfection. G55TL and HGBM were selected with 6 μg ml^−1^, G141 with 4 μg ml^−1^ to obtain resistant polyclonal cell pools. Gene expression analysis was performed using western blot and qPCR analyses.

### Tumour xenografts and tumour volume measurement

Xenograft transplantations were performed in a nonblinded manner in nonrandomized 6- to 7-week-old female athymic NMRI nu/nu mice according to the institutional guidelines and permissions for animal experiments, obtained from the regional authorities of the State of Hessen. In all, 5,000 cells (all G55TL-derived transgenic cell pools), 25,000 cells (PHD3-/- and control murine astrocytomas), 20,000 cells (PHD3 WT and PHD3 hydroxylase-deficient mutant astrocytoma rescue experiments) in a volume of 1 μl, or 200,000 cells (GBM046x-derived pools) in a volume of 2 μl, were stereotactically implanted intracranially into the left striatum. Group size was chosen based on previous empirical experience with analogous animal tumour models^23^,^52^. After the onset of neurological symptoms all mice were killed at the same time point. For drug treatment experiments, mice that developed premature morbidity were killed and excluded from the experiment. Mice were anaesthetized with Ketamine and Xylazine. The chest was opened and the vasculature was perfused with 0.9% NaCl solution for 2 min and fixative (4% paraformaldehyde, PFA) for 6 min. Brains were removed and additionally fixed overnight in 4% dehydrated in 30% sucrose and rapidly frozen on dry ice for sectioning with a cryotome. The sections were stained with haematoxylin and eosin and tumour volume was determined using stereological quantification of series of every sixth 40-μm sections (240 μm intervals, for G55 tumours) or every twelfth 40-μm sections (480 μm intervals for mouse astrocytoma and GBM046x tumours) throughout the brain. The tumour area was traced with a semiautomatic stereology system (MicroBrightField) or measured on light micrographs using ImageJ.

For subcutaneous tumour experiments, 50,000 G55 cells suspended in 0.1 ml PBS were injected subcutaneously into the flanks of 6- to 7-week-old female nude (NMRI nu/nu) mice. Mice were given 100 mg kg^−1^ Erlotinib/10% dimethylsulphoxide (DMSO) in saline or 10% DMSO in saline solution orally every day. The tumour size was measured every 2 days with a caliper and the tumour volume was calculated using the formula: tumour volume=0.52 × *d*^2^ × *D* (where *d* is the short tumour diameter and *D* is the long tumour diameter).

### Vessel density, cell apoptosis and proliferation *in vivo*

For immunohistochemical detection of vessel density, free-floating intracranial tumour sections were washed in PBS. Endogenous peroxidase was neutralized with 0.6% H_2_O_2_ in PBS for 30 min. After washing again in PBS, sections were mounted on microscope slides and dried for 3 h at room temperature (RT). Antigen retrieval was performed for 5 min in Tris-EDTA buffer, pH 8.0, in a steamer. Sections were blocked with 20% normal goat serum (NGS)/0.01% Triton in PBS for 2 h. Sections were then treated overnight at 4 °C with CD34 primary antibody (1:100, BD Pharmingen) in 10% NGS/0.01% Triton in PBS, respectively. After washing in PBS, sections were incubated with a secondary peroxidase-conjugated goat anti-rat IgG antibody, and diluted 1:200 in 10% NGS/0.01% Triton in PBS for 1 h. After a PBS wash, visualization was performed using the CSA II, Biotin-Free Catalysed Amplification System (Dako) according to the manufacturer’s instructions. After washing in PBS, sections were counterstained in haematoxylin (1:5 diluted in distilled water) for 8 min and mounted in Entellan or Aquatex (Merck Millipore). Vascular density of tumours was quantified by measuring blood vessel area stained with CD34 in 10 randomly chosen optical fields ( × 20) per tumour. For quantification of apoptosis *in vivo*, tumour-bearing brain sections were washed in PBS, mounted on microscope slides and dried overnight at RT, followed by antigen retrieval for 5 min in 0.01 M Sodium Citrate buffer (pH 6). TUNEL staining was performed using the ApopTag Plus Fluorescein In Situ Apoptosis Detection Kit (Chemicon, Hampshire, UK) with alternative use of alkaline phosphatase-conjugated anti-digoxigenin antibody (1:500, Roche Diagnostics), NBT/BCIP (Roche Diagnostics)-based color development and subsequent counterstain with Nuclear Fast Red for 3–5 min. For immunohistochemical analysis of cell proliferation *in vivo*, sections were hydrated in PBS and incubated for 30 min in 0.6% H_2_O_2_/PBS. After washing in PBS (three times, 5 min), brain sections were mounted on slides and tried for 3 h overnight at RT, followed by boiling for 5 min in TE buffer (pH 8.0) for antigen retrieval. Slides were cooled down for 15 min at RT washed in PBS, blocked for 2 h (4% NGS/5% BSA/0.01% Triton X-100/PBS) and incubated with Phospho-Histone H3 primary antibody (1:100, no. IHC-00061, Bethyl Laboratories Inc.) in blocking buffer overnight at 4 °C. The following day sections were washed in PBS and anti-rabbit N-Histofine (no. 414141, Nichirei Bioscience Inc.) was applied for 1 h incubation at RT followed by PBS washing. Colour development was performed by incubating section with diaminobenzidine solution (DCS Innovative Diagnostik-Systeme or Dako). After washing in PBS, sections were counterstained in haematoxylin (1:5–1:10 diluted in distilled water) for 8 min, dehydrated with ascending alcohol series and mounted in Entellan (Merck Millipore). TUNEL or phospho-histone H3-positive cells were quantified using an automated procedure employing colour thresholding and particle analysis in up to 15 high-magnification non-necrotic optical fields per tumour.

### Sphere-forming units and *in vitro* growth

For quantification of sphere-forming units, cells were seeded at 500–1,000 cells per well in pHEMA (10 mg ml^−1^ poly(2-hydroxyethyl methacrylate in 95% ethanol) coated six-well suspension culture plates (*n*=6). The cells were incubated in Neurosphere medium (see above)±the addition of growth factors and where applicable respective reagents. Ninety-six hours later (for GBM046x cells—7 days later) spheres were counted and the percentage of sphere-forming cells was calculated. Cell accumulation was assessed by seeding cells at 100,000 cells per 10-cm dish in B27/F12 medium±the addition of EGF/FGF in normoxia or 1% hypoxia for 72 h (*n*=6).

### Cell apoptosis and proliferation *in vitro*

To detect cell apoptosis, cells were seeded at 400,000 cells per 10-cm dish and incubated for 24 h in F12 medium ± EGF/FGF. In all, 250,000 cells were transferred to slides using a Cyto-Tek Centrifuge (Sakura Finetek) after dissociation with accutase. TUNEL staining was performed using the ApopTag Plus Fluorescein In Situ Apoptosis Detection Kit (Chemicon). Apoptosis was quantified as the percentage of TUNEL-positive cells in five randomly selected optical fields ( × 20) per condition.

### Immunofluorescence

In all, 200,000 cells were seeded on pHEMA-coated 6-cm dishes and cultivated as tumour spheres. For p65 staining, spheres were stimulated for 30 min with 10 ng ml^−1^ TNF-α. Spheres were transferred on a slide using a Cyto-Tek Centrifuge (Sakura Finetek). Fixation was performed in 4% PFA for 10 min at 4 °C followed by a rinse in PBS (two times, 2 min). The spheres were permeabilized with 0.1% Triton X-100 in PBS for 4 min on ice, washed for 5 min with PBS and blocking solution (PBS/0.1% Triton X-100/1% BSA/4% donkey serum) was applied. Then, spheres were incubated with rabbit anti-NF-κB p65 (1:200 in blocking solution, Cell Signalling; D14E12, no. 8242) overnight at 4 °C. After extensive washing with PBS, secondary anti-rabbit Cy3 (1:200 in blocking solution) and Phallodin-fluorescein isothiocyanate (FITC; 1:250 in blocking solution) were added for 1 h at RT. Subsequently, the slides were washed once with PBS, followed by incubation with DAPI (1:1,000 in PBS) for 10 min. After a final wash with PBS, the slides were mounted using DAKO mounting medium.

### Luciferase reporter assay

Cells were transiently transfected with an NF-κB-responsive^7^ promoter firefly and a SV-40-Renilla luciferase construct (Promega) for normalization of transfection efficiency, grown for 48 h under sphere conditions and assayed for luciferase activity with the Dual-Luciferase Reporter-Assay System (Promega).

### Real-time qRT–PCR

RNA was extracted with the RNeasy Mini Kit (Qiagen), and reverse-transcribed using standard protocols (Superscript II Reverse Transcriptase, Life Technologies or RevertAid H Minus M-MuLV Reverse Transcriptase, Fermentas). cDNA was amplified using the ABsolute QPCR SYBR Green Mix or the ABsolute QPCR Mix (ABgene; primer and probe sequences have been previously described^19^). Gene-specific PCR products were measured continuously in an iCycler iQ-Systems (Bio-Rad) or StepOnePlus real-time PCR system (Applied Biosystems) for up to 45 cycles. The difference in the threshold number of cycles between the gene of interest and hypoxanthine phosphoribosyltransferase 1 was then normalized relative to the standard chosen for each experiment and converted into fold difference.

### Immunoblotting

For immunoblotting cells were harvested in PBS (4 °C), cell pellets were lysed in 10 mM Tris/HCl (pH 7,5), 2% SDS, 2 mM EGTA, 20 mM NaF or in 50 mM Tris/HCl buffer (pH 7.5), 1% Triton X-100, 150 mM NaCl, 10 mM sodium pyrophosphate, 20 mM NaF, 1 mM sodium orthovanadate and 1% complete protease inhibitor cocktail (Roche). Protein (25 μg) lysates were subjected to SDS–PAGE and western blot analysis was performed using antibodies specific for HIF-1α (BD Transduction Laboratories, no. 610958), HIF-2α, PHD1, PHD2, PHD3 (Novus Biologicals, NB 100-122, NB-100-310, NB100-137 and NB-100-303, respectively), pEGFR (Tyr 1173; Santa Cruz Biotechnology), pEGFR (Tyr 1068; Cell Signalling), EGFR (Cell Signalling or Millipore) and tubulin as a loading control (Jackson Lab, DLN09992 or Invitrogen). Immunoreactive bands were detected with horse radish peroxidase (HRP)-conjugated secondary antibodies through enhanced chemiluminescence (ECL, Thermo, Perkin-Elmer and GE Healthcare).

### Phospho-RTK array analysis

The phosphorylation level of 42 human RTKs was assessed using the Proteome Profiler Human Phospho-RTK Array Kit (R&D Systems). 350,000 cells were plated and cultivated for 48 h in Neurosphere medium without EGF/FGF. Cells were lysed in NP-40 Buffer (1% NP-40, 20 mM Tris-HCl, pH 8.0, 137 mM NaCl, 10% glycerol, 2 mM EDTA, 1 mM sodium orthovanadate, 10 μg ml^−1^ Aprotinin, 10 μg ml^−1^ Leupeptin). 150 μg of total protein were incubated with RTK array membranes. Detection of proteins was accomplished using mouse anti-phospho-tyrosine antibody conjugated to HRP and enhanced chemiluminescence, according to the manufacturer’s instructions.

### PHD3 promoter methylation analyses

Cells were incubated from the second day on for 72 h with 5-AzaC (20 μM), which was changed every day (for demethylation). The last 24 h, TSA (1 μM) was additionally added ±hypoxic treatment at 1% O_2_. DNA was isolated from the cells or tissue using commercial kits (DNeasy Blood&Tissue Kit no. 69506 and QIAamp DNA FFPE Tissue Kit no. 56404, Qiagen). CpGenome Universal methylated DNA (Chemicon/Millipore no. S 7821) served as a positive control, DNA isolated from blood with the Blood & Cell Culture DNA Spin Kit (Genomed no. 440250) was used as a negative control. DNA was then subjected to sodium bisulphite modification using the EZ DNA methylation kit (EZ DNA Methylation-Gold Kit no. D5006 Zymo Research). To assess the methylation status of the PHD3-associated 5′-CpG island, methylation-specific PCR was carried out. Two sets of primers (see primer sequences) detecting either methylated or unmethylated sequences were used with slight modifications^16^ (see Supplementary Methods: Primer and shRNA sequences). Methylation-specific PCR was performed using Qiagen HotStar DNA Polymerase in a reaction volume of 25 μl. The PCR conditions were as described in ref. 16. The PCR reaction mixture was loaded on a 2% agarose gel, stained with ethidium bromide and visualized under UV light.

### Bioinformatic analysis

PHD3 gene expression data (Agilent 244 K Custom Gene Expression G4502A-07 array, log 2 tumour/normal ratio, total of 424 samples with available expression data) and PHD3 promoter methylation data (Illumina Infinium Human DNA Methylation 27 array, probe cg05769169, total of 251 samples with available methylation data) for the glioblastoma cohort of The Cancer Genome Atlas (TCGA)^21^ were downloaded from the TCGA data portal ( http://tcga-portal.nci.nih.gov/tcga-portal/AnomalySearch.jsp) on 7 May 2012. EGFR and PHD3 copy number alteration (CNA) data for the TCGA cohort, determined using the GISTIC algorithm (total of 501 samples with available CNA data) were downloaded using the WebAPI of the cBio portal ( http://www.cbioportal.org/public-portal/web_api.jsp)^53^ on 9 September 2012. For PHD3 promoter methylation analysis, tumours with a beta-value>0.1 for probe cg05769169 were considered as methylated, tumours with a beta-value ≤0.1 were considered as unmethylated. CNAs along chromosome 14 were determined using GISTIC analysis at the TCGA Copy Number Portal ( http://www.broadinstitute.org/tcga/home) and the Integrative Genomics Viewer^54^.

### Statistical analysis

Results are presented as mean±s.e.m. Statistical analysis was performed using the Student’s *t*-test or, in the case of multiple comparisons in the glioma patient cohort, the ANOVA test followed by a post test for linear trend. The Grubbs test was used to eliminate outliers for the expression of CAIX and PHD3 and for *in vivo* tumour growth experiments. Statistical significance was defined as *P*<0.05 (*P<0.05; **P<0.01, ***P<0.001).

## Author contributions

A.-T.H. generated and biochemically and functionally characterized knockdown and overexpression cell pools, analysed xenograft experiments, performed immunostainings and qPCR. B.K.G. collected data and performed bioinformatic analysis. S.S. performed transplantations and analysed tumour volume, carried out *in vivo* and *in vitro* proliferation and apoptosis assays. A.M.C. performed *in vivo* tumour experiments and drug treatment. M.R. and A.F. generated stable cell lines. F.F. performed immunofluorescence and biochemistry. H.D. and C.L.E. performed biochemistry. P.H.M. and P.C. generated the conditional PHD3-/- mice. G.R. provided and characterized the grade II–IV glioma collection. T.A., A.A.-P. and B.K.G. designed experiments, analysed the data, prepared the figures and wrote the manuscript. All authors discussed the results and commented on the manuscript.

## Additional information

**How to cite this article:** Henze, A.-T. *et al.* Loss of PHD3 allows tumours to overcome hypoxic growth inhibition and sustain proliferation through EGFR. *Nat. Commun.* 5:5582 doi: 10.1038/ncomms6582 (2014).

## Supplementary Material

Supplementary Information Supplementary Figures 1-13, Supplementary Methods and Supplementary References

## References

[b1] Goda N. *et al.* Hypoxia-inducible factor 1alpha is essential for cell cycle arrest during hypoxia. Mol. Cell Biol. 23, 359–369 (2003). 12482987 10.1128/MCB.23.1.359-369.2003 PMC140666

[b2] Bertout J. A., Patel S. A. & Simon M. C. The impact of O2 availability on human cancer. Nat. Rev. Cancer 8, 967–975 (2008). 18987634 10.1038/nrc2540 PMC3140692

[b3] Bruick R. K. & McKnight S. L. A conserved family of prolyl-4-hydroxylases that modify HIF. Science 294, 1337–1340 (2001). 11598268 10.1126/science.1066373

[b4] Epstein A. C. *et al.* C. elegans EGL-9 and mammalian homologs define a family of dioxygenases that regulate HIF by prolyl hydroxylation. Cell 107, 43–54 (2001). 11595184 10.1016/s0092-8674(01)00507-4

[b5] Kaelin W. G. Jr. & Ratcliffe P. J. Oxygen sensing by metazoans: the central role of the HIF hydroxylase pathway. Mol. Cell 30, 393–402 (2008). 18498744 10.1016/j.molcel.2008.04.009

[b6] Chan D. A. *et al.* Tumor vasculature is regulated by PHD2-mediated angiogenesis and bone marrow-derived cell recruitment. Cancer Cell 15, 527–538 (2009). 19477431 10.1016/j.ccr.2009.04.010 PMC2846696

[b7] Cummins E. P. *et al.* Prolyl hydroxylase-1 negatively regulates IkappaB kinase-beta, giving insight into hypoxia-induced NFkappaB activity. Proc. Natl Acad. Sci. USA 103, 18154–18159 (2006). 17114296 10.1073/pnas.0602235103 PMC1643842

[b8] Köditz J. *et al.* Oxygen-dependent ATF-4 stability is mediated by the PHD3 oxygen sensor. Blood 110, 3610–3617 (2007). 17684156 10.1182/blood-2007-06-094441

[b9] Luo W. *et al.* Pyruvate kinase M2 is a PHD3-stimulated coactivator for hypoxia-inducible factor 1. Cell 145, 732–744 (2011). 21620138 10.1016/j.cell.2011.03.054 PMC3130564

[b10] Xie L. *et al.* Oxygen-regulated beta(2)-adrenergic receptor hydroxylation by EGLN3 and ubiquitylation by pVHL. Sci. Signal. 2, ra33 (2009). 19584355 10.1126/scisignal.2000444 PMC2788937

[b11] Xue J. *et al.* Prolyl hydroxylase-3 is down-regulated in colorectal cancer cells and inhibits IKKbeta independent of hydroxylase activity. Gastroenterology 138, 606–615 (2010). 19786027 10.1053/j.gastro.2009.09.049

[b12] Erez N. *et al.* Expression of prolyl-hydroxylase-1 (PHD1/EGLN2) suppresses hypoxia inducible factor-1alpha activation and inhibits tumor growth. Cancer Res. 63, 8777–8783 (2003). 14695194

[b13] Zhang Q. *et al.* Control of cyclin D1 and breast tumorigenesis by the EglN2 prolyl hydroxylase. Cancer Cell 16, 413–424 (2009). 19878873 10.1016/j.ccr.2009.09.029 PMC2788761

[b14] Koivunen P. *et al.* Transformation by the (R)-enantiomer of 2-hydroxyglutarate linked to EGLN activation. Nature 483, 484–488 (2012). 22343896 10.1038/nature10898 PMC3656605

[b15] Mazzone M. *et al.* Heterozygous deficiency of PHD2 restores tumor oxygenation and inhibits metastasis via endothelial normalization. Cell 136, 839–851 (2009). 19217150 10.1016/j.cell.2009.01.020 PMC4037868

[b16] Hatzimichael E. *et al.* The prolyl-hydroxylase EGLN3 and not EGLN1 is inactivated by methylation in plasma cell neoplasia. Eur. J. Haematol. 84, 47–51 (2010). 19737309 10.1111/j.1600-0609.2009.01344.x

[b17] Sciorra V. A., Sanchez M. A., Kunibe A. & Wurmser A. E. Suppression of glioma progression by Egln3. PLoS ONE 7, e40053 (2012). 22905089 10.1371/journal.pone.0040053 PMC3414484

[b18] Su Y. *et al.* PHD3 regulates differentiation, tumour growth and angiogenesis in pancreatic cancer. Br. J. Cancer. 103, 1571–1579 (2010). 20978507 10.1038/sj.bjc.6605936 PMC2990580

[b19] Henze A. T. *et al.* Prolyl hydroxylases 2 and 3 act in gliomas as protective negative feedback regulators of hypoxia-inducible factors. Cancer Res. 70, 357–366 (2010). 20028863 10.1158/0008-5472.CAN-09-1876

[b20] Lee S. *et al.* Neuronal apoptosis linked to EglN3 prolyl hydroxylase and familial pheochromocytoma genes: developmental culling and cancer. Cancer Cell 8, 155–167 (2005). 16098468 10.1016/j.ccr.2005.06.015

[b21] The Cancer Genome Atlas Research Network. Comprehensive genomic characterization defines human glioblastoma genes and core pathways. Nature 455, 1061–1068 (2008). 18772890 10.1038/nature07385 PMC2671642

[b22] Toedt G. *et al.* Molecular signatures classify astrocytic gliomas by IDH1 mutation status. Int. J. Cancer 128, 1095–1103 (2011). 20473936 10.1002/ijc.25448

[b23] Sawamiphak S. *et al.* Ephrin-B2 regulates VEGFR2 function in developmental and tumour angiogenesis. Nature 465, 487–491 (2010). 20445540 10.1038/nature08995

[b24] Hanahan D. & Weinberg R. A. Hallmarks of cancer: the next generation. Cell 144, 646–674 (2011). 21376230 10.1016/j.cell.2011.02.013

[b25] Fu J. & Taubman M. B. Prolyl hydroxylase EGLN3 regulates skeletal myoblast differentiation through an NF-kappaB-dependent pathway. J. Biol. Chem. 285, 8927–8935 (2010). 20089853 10.1074/jbc.M109.078600 PMC2838314

[b26] Helin K. *et al.* The biological activity of the human epidermal growth factor receptor is positively regulated by its C-terminal tyrosines. Oncogene 6, 825–832 (1991). 1646987

[b27] Nicholas M. K., Lukas R. V., Jafri N. F., Faoro L. & Salgia R. Epidermal growth factor receptor—mediated signal transduction in the development and therapy of gliomas. Clin. Cancer Res. 12, 7261–7270 (2006). 17189397 10.1158/1078-0432.CCR-06-0874

[b28] Fraisl P., Aragones J. & Carmeliet P. Inhibition of oxygen sensors as a therapeutic strategy for ischaemic and inflammatory disease. Nat. Rev. Drug Discov. 8, 139–152 (2009). 19165233 10.1038/nrd2761

[b29] Acker T. *et al.* Genetic evidence for a tumor suppressor role of HIF-2alpha. Cancer Cell 8, 131–141 (2005). 16098466 10.1016/j.ccr.2005.07.003

[b30] Blancher C., Moore J. W., Talks K. L., Houlbrook S. & Harris A. L. Relationship of hypoxia-inducible factor (HIF)-1alpha and HIF-2alpha expression to vascular endothelial growth factor induction and hypoxia survival in human breast cancer cell lines. Cancer Res. 60, 7106–7113 (2000). 11156418

[b31] Carmeliet P. *et al.* Role of HIF-1alpha in hypoxia-mediated apoptosis, cell proliferation and tumour angiogenesis. Nature 394, 485–490 (1998). 9697772 10.1038/28867

[b32] Hubbi M. E., Luo W., Baek J. H. & Semenza G. L. MCM proteins are negative regulators of hypoxia-inducible factor 1. Mol. Cell 42, 700–712 (2011). 21658608 10.1016/j.molcel.2011.03.029 PMC3131976

[b33] Liu L. *et al.* Hypoxia-induced energy stress regulates mRNA translation and cell growth. Mol. Cell 21, 521–531 (2006). 16483933 10.1016/j.molcel.2006.01.010 PMC3153113

[b34] Rantanen K. *et al.* Prolyl hydroxylase PHD3 activates oxygen-dependent protein aggregation. Mol. Biol. Cell 19, 2231–2240 (2008). 18337469 10.1091/mbc.E07-11-1124 PMC2366864

[b35] Schlisio S. *et al.* The kinesin KIF1Bbeta acts downstream from EglN3 to induce apoptosis and is a potential 1p36 tumor suppressor. Genes Dev. 22, 884–893 (2008). 18334619 10.1101/gad.1648608 PMC2279200

[b36] Tennant D. A. & Gottlieb E. HIF prolyl hydroxylase-3 mediates alpha-ketoglutarate-induced apoptosis and tumor suppression. J. Mol. Med. (Berl) 88, 839–849 (2010). 20383689 10.1007/s00109-010-0627-0

[b37] Bishop T. *et al.* Abnormal sympathoadrenal development and systemic hypotension in PHD3-/- mice. Mol. Cell Biol. 28, 3386–3400 (2008). 18332118 10.1128/MCB.02041-07 PMC2423159

[b38] Darby C., Cosma C. L., Thomas J. H. & Manoil C. Lethal paralysis of *Caenorhabditis elegans* by *Pseudomonas aeruginosa*. Proc. Natl Acad. Sci. USA 96, 15202–15207 (1999). 10611362 10.1073/pnas.96.26.15202 PMC24797

[b39] Appelhoff R. J. *et al.* Differential function of the prolyl hydroxylases PHD1, PHD2, and PHD3 in the regulation of hypoxia-inducible factor. J. Biol. Chem. 279, 38458–38465 (2004). 15247232 10.1074/jbc.M406026200

[b40] del Peso L. *et al.* The von Hippel Lindau/hypoxia-inducible factor (HIF) pathway regulates the transcription of the HIF-proline hydroxylase genes in response to low oxygen. J. Biol. Chem. 278, 48690–48695 (2003). 14506252 10.1074/jbc.M308862200

[b41] Lipscomb E. A., Sarmiere P. D. & Freeman R. S. SM-20 is a novel mitochondrial protein that causes caspase-dependent cell death in nerve growth factor-dependent neurons. J. Biol. Chem. 276, 5085–5092 (2001). 11060309 10.1074/jbc.M008407200

[b42] Huang K. T., Mikeska T., Dobrovic A. & Fox S. B. DNA methylation analysis of the HIF-1alpha prolyl hydroxylase domain genes PHD1, PHD2, PHD3 and the factor inhibiting HIF gene FIH in invasive breast carcinomas. Histopathology 57, 451–460 (2010). 20727020 10.1111/j.1365-2559.2010.03633.x

[b43] Place T. L. *et al.* Aberrant promoter CpG methylation is a mechanism for impaired PHD3 expression in a diverse set of malignant cells. PLoS ONE 6, e14617 (2011). 21297970 10.1371/journal.pone.0014617 PMC3030558

[b44] Rawluszko A. A., Bujnicka K. E., Horbacka K., Krokowicz P. & Jagodzi Ski P. P. Expression and DNA methylation levels of prolyl hydroxylases PHD1, PHD2, PHD3 and asparaginyl hydroxylase FIH in colorectal cancer. BMC Cancer 13, 526 (2013). 24195777 10.1186/1471-2407-13-526 PMC3828400

[b45] Baek J. H. *et al.* OS-9 interacts with hypoxia-inducible factor 1alpha and prolyl hydroxylases to promote oxygen-dependent degradation of HIF-1alpha. Mol. Cell 17, 503–512 (2005). 15721254 10.1016/j.molcel.2005.01.011

[b46] Fu J., Menzies K., Freeman R. S. & Taubman M. B. EGLN3 prolyl hydroxylase regulates skeletal muscle differentiation and myogenin protein stability. J. Biol. Chem. 282, 12410–12418 (2007). 17344222 10.1074/jbc.M608748200

[b47] Hopfer U., Hopfer H., Jablonski K., Stahl R. A. & Wolf G. The novel WD-repeat protein Morg1 acts as a molecular scaffold for hypoxia-inducible factor prolyl hydroxylase 3 (PHD3). J. Biol. Chem. 281, 8645–8655 (2006). 16407229 10.1074/jbc.M513751200

[b48] Masson N. *et al.* The HIF prolyl hydroxylase PHD3 is a potential substrate of the TRiC chaperonin. FEBS Lett. 570, 166–170 (2004). 15251459 10.1016/j.febslet.2004.06.040

[b49] Garvalov B. K. *et al.* PHD3 regulates EGFR internalization and signalling in tumours. Nat. Commun **5**:5577 doi: 10.1038/ncomms6577 (2014). 10.1038/ncomms6577 25420589

[b50] Blouw B. *et al.* The hypoxic response of tumors is dependent on their microenvironment. Cancer Cell 4, 133–146 (2003). 12957288 10.1016/s1535-6108(03)00194-6

[b51] Louis D. N. *et al.* The 2007 WHO classification of tumours of the central nervous system. Acta Neuropathol. 114, 97–109 (2007). 17618441 10.1007/s00401-007-0243-4 PMC1929165

[b52] Seidel S. *et al.* A hypoxic niche regulates glioblastoma stem cells through hypoxia inducible factor 2α. Brain 133, 983–995 (2010). 20375133 10.1093/brain/awq042

[b53] Cerami E. *et al.* The cBio cancer genomics portal: an open platform for exploring multidimensional cancer genomics data. Cancer Discov. 2, 401–404 (2012). 22588877 10.1158/2159-8290.CD-12-0095 PMC3956037

[b54] Robinson J. T. *et al.* Integrative genomics viewer. Nat. Biotechnol. 29, 24–26 (2011). 21221095 10.1038/nbt.1754 PMC3346182

