# Supplementary material for: Loss of PHD3 allows tumours to overcome hypoxic growth inhibition and sustain proliferation through EGFR
Source: Nat Commun. 2014 Nov 25;5:5582. doi: 10.1038/ncomms6582 (PMC4263145; doi:10.1038/ncomms6582)
Supplement: Supplementary Information — Supplementary Figures 1-13, Supplementary Methods and Supplementary References [file ncomms6582-s1.pdf]

Supplementary Information

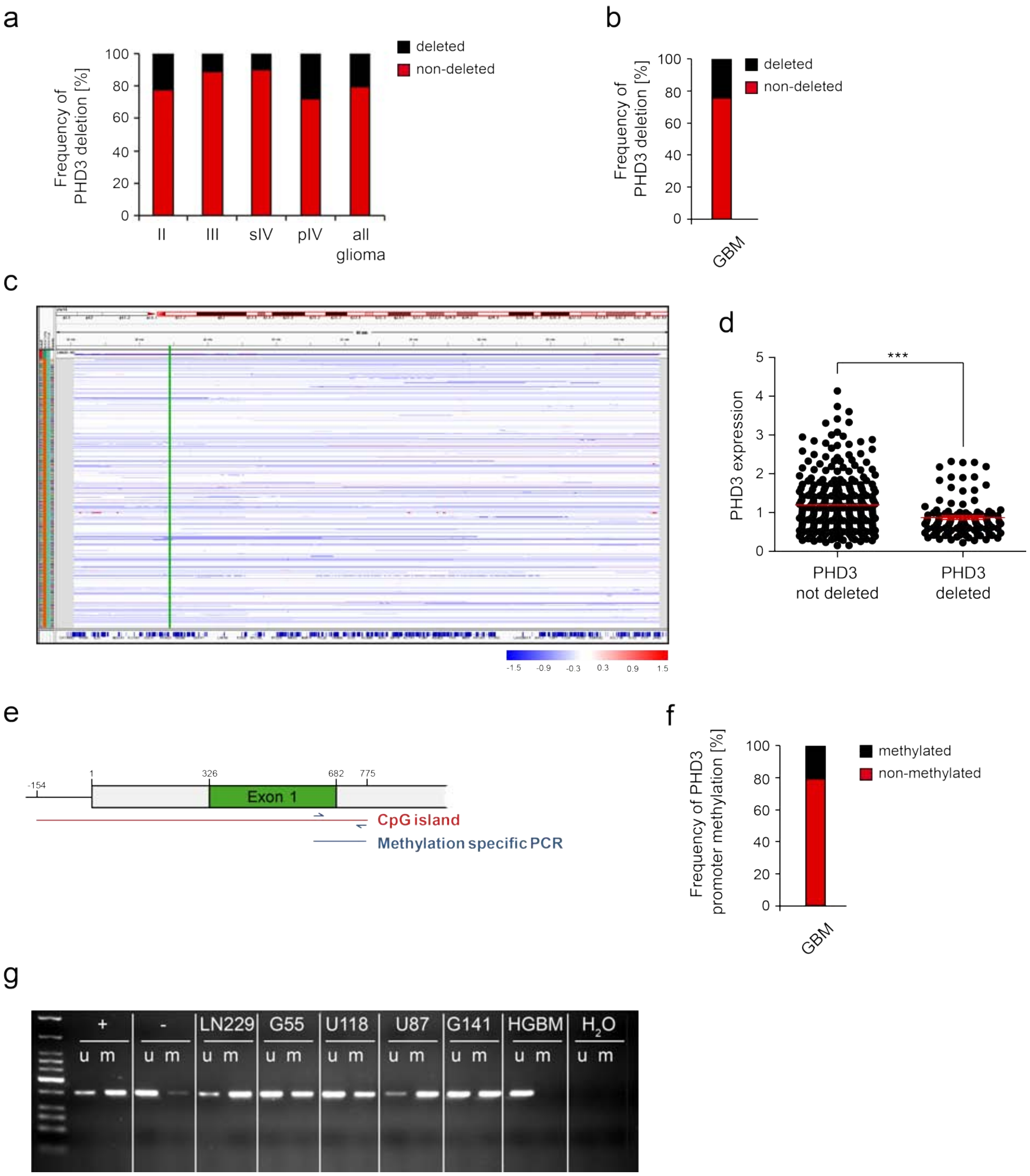

Supplementary Figure 1 │ Genetic and epigenetic alterations of PHD3 expression

a, PHD3 is deleted in a subset of gliomas of all WHO grades. Fraction of tumours with PHD3 deletion in gliomas of different WHO grades as determined by PHD3 gene copy number analysis using array comparative genomic hybridization[1]. No PHD3 amplification was

detected in any of the tumours, including diffuse astrocytomas (WHO grade II), anaplastic astrocytomas (WHO grade III), (s)econdary and (p)rimary glioblastomas (WHO grade IV). **b,** Fraction of glioblastomas with PHD3 deletion in the TCGA glioblastoma cohort. **c,** Distribution of copy number alterations along chromosome 14q. Gene dosages as determined by the GISTIC 2.0 algorithm are mapped for each tumour (horizontal lines) according to the gene order along chromosome 14q; the cutoff for deletion (represented in blue) is -0.3. The position of the PHD3 mRNA coding sequence is indicated by the vertical green line. **d,** PHD3 deletion is associated with reduced PHD3 expression. The expression of PHD3 was plotted for tumours with or without PHD3 deletion in the TCGA glioblastoma cohort. **e,** Diagram of the CpG island in the PHD3 promoter region, with the locations of the methylation specific primers indicated. **f,** Fraction of tumours with PHD3 promoter methylation in the TCGA glioblastoma cohort. **g,** Differential methylation of the PHD3 promoter CpG island in glioblastoma cell lines. A panel of glioblastoma cell lines was analyzed for the presence of unmethylated (u) and methylated (m) PHD3 promoter sequences by methylation-specific PCR. The PHD3 promoter is methylated in most glioblastoma cell lines, but not in the HGBM line. "+" and "-" are methylated and unmethylated controls, respectively.

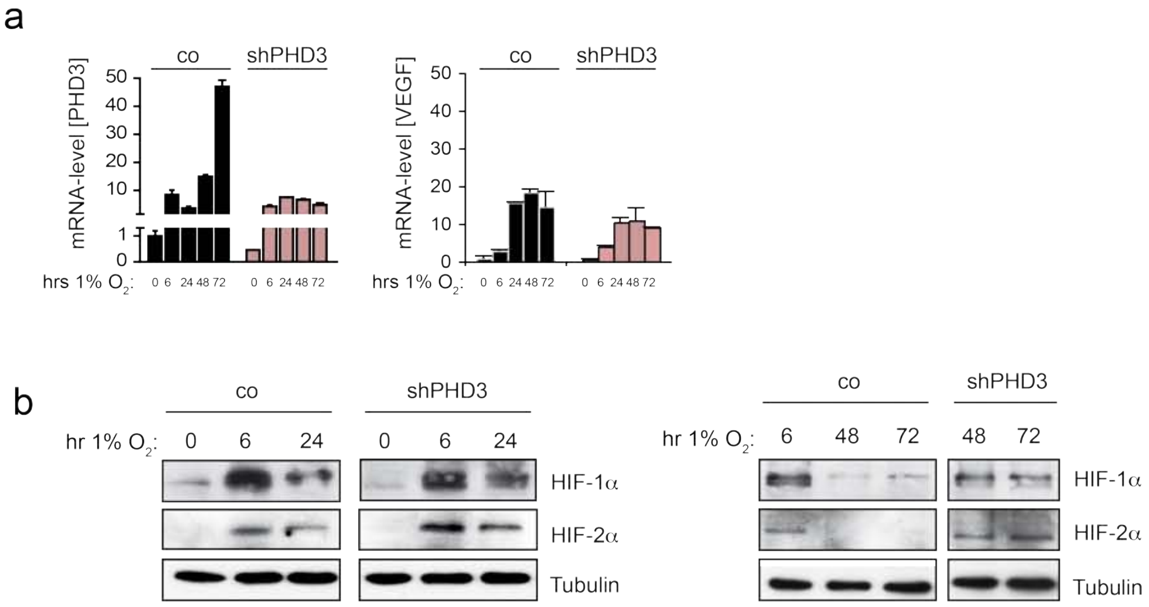

Supplementary Figure 2 │ Characterization of glioblastoma PHD3 knockdown cell pools

a, PHD3 and VEGF mRNA quantification by qPCR in G55 glioblastoma cells stably transduced with control (co) or PHD3 shRNA and exposed to 21% (0 hr) or to 1% O_2 for the indicated times. Values are normalized to the housekeeping gene HPRT and expressed as fold induction relative to normoxic control (n=2). **b,** PHD3 knockdown results in accumulation of HIF-1α and HIF-2α during chronic hypoxia. Immunoblot of G55 cells stably transduced with control (co) or PHD3 shRNA following exposure to 21% or 1% O_2 for the indicated times. Western Blots images (**b**) have been cropped for presentation. Full size images are presented in Supplementary Fig. 11.

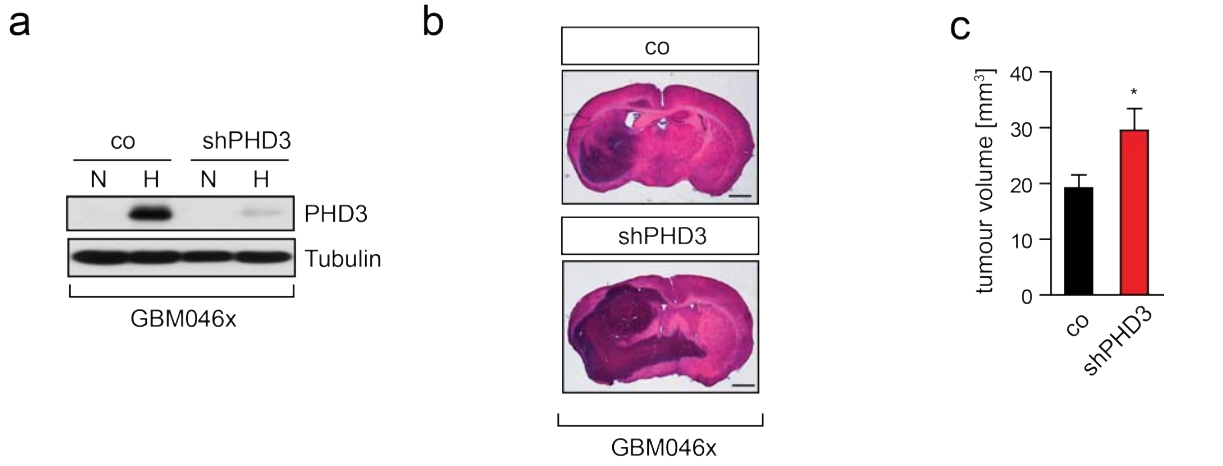

Supplementary Figure 3 │ PHD3 loss increases glioblastoma growth

a, Immunoblot of the primary glioblastoma line GBM046x stably transduced with control (co) or PHD3 shRNA, following exposure to 21% (N) or 1% O_2 (H) for 48 h. **b, c,** PHD3 loss promotes intracranial glioma growth. Tumour xenografts of GBM046x cells expressing control or PHD3 shRNA were stained with haematoxylin and eosin (HE) (n=8) (**b**) and tumour growth was quantified (**c**). Western Blots images (**a**) have been cropped for presentation. Full size images are presented in Supplementary Fig. 11. Scale bars, 1 mm.

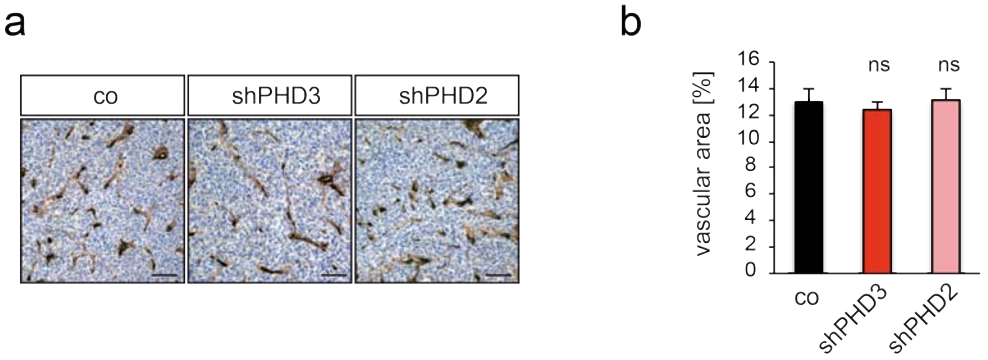

Supplementary Figure 4 │ PHD3 loss does not affect vessel density

a, b, Vessel density assessed by CD34 staining is not altered in intracranial tumours generated by G55 cells with PHD3 or PHD2 silencing (n=8) (**a**). Vessel density was quantified as the percentage of total tumour area occupied by vessels (**b**). All values are means + SEM, n.s., non-significant. Scale bars, 50 µm.

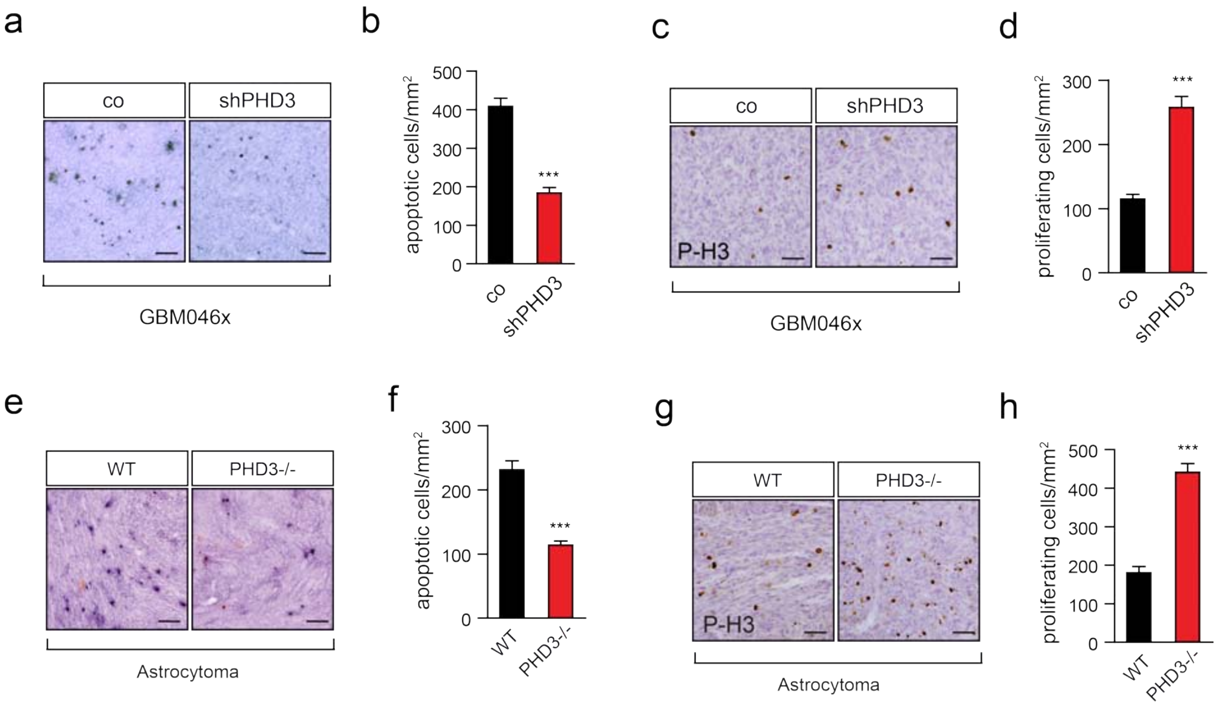

Supplementary Figure 5 │ Loss of PHD3 decreases tumour apoptosis and increases proliferation

a-d, PHD3 loss reduces tumour cell apoptosis (**a**, **b**) and increases cell proliferation (**c, d**) in xenografts of GBM046x cells (n=8). Apoptosis and proliferation were assessed by quantifying the number of TUNEL positive cells and the number of phospho-histone 3 positive cells per tumour area, respectively. **e-h,** PHD3 loss reduces tumour cell apoptosis (**e, f**) and increases proliferation (**g, h**) in xenografts of astrocytomas (n=9-10). Apoptosis and proliferation were assessed as described above (**a-d**). All values are means + SEM, * $p<0.05$; ** $p<0.01$; *** $p<0.001$. Scale bars, 50 µm.

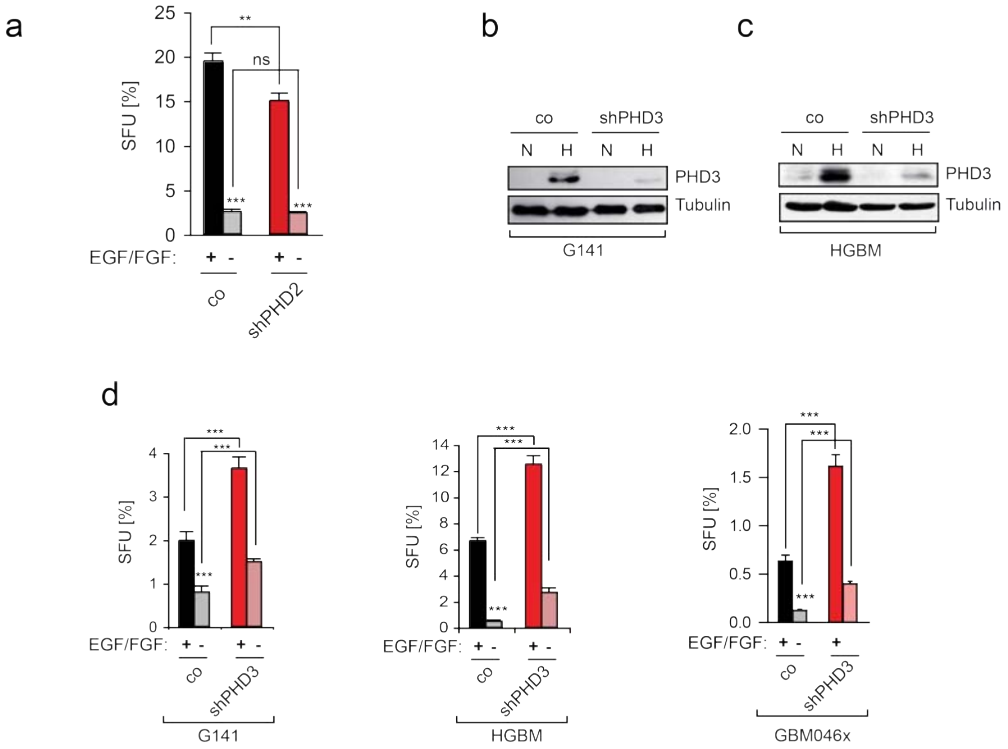

Supplementary Figure 6 │ Loss of PHD3 but not PHD2 promotes tumour cell survival

a, PHD2 loss does not increase clonal cell growth in a three-dimensional spheroid culture system. G55 cells expressing control or PHD2 shRNA were cultured as spheroids in B27 supplemented serum-free medium ± EGF/FGF and the number of spheroids was quantified after 3 days (n=6). **b, c,** Immunoblot of G141 and HGBM cells stably transduced with control (co) or PHD3 shRNA following exposure to 21% (N) or 1% O_2 (H) for 24 h. **d,** PHD3 disruption by shRNA confers a growth advantage in additional glioma cell systems. Clonal cell growth was quantified in the presence or absence of EGF/FGF by counting the number of spheroids after 3 days in the glioblastoma lines G141 and HGBM (n=6) and in the primary glioblastoma line GBM046x (n=6). Western Blots images (**b, c**) have been cropped for presentation. Full size images are presented in Supplementary Fig. 11. All values are means + SEM, ** p<0.01; ***p<0.001. Scale bars, 50 μm.

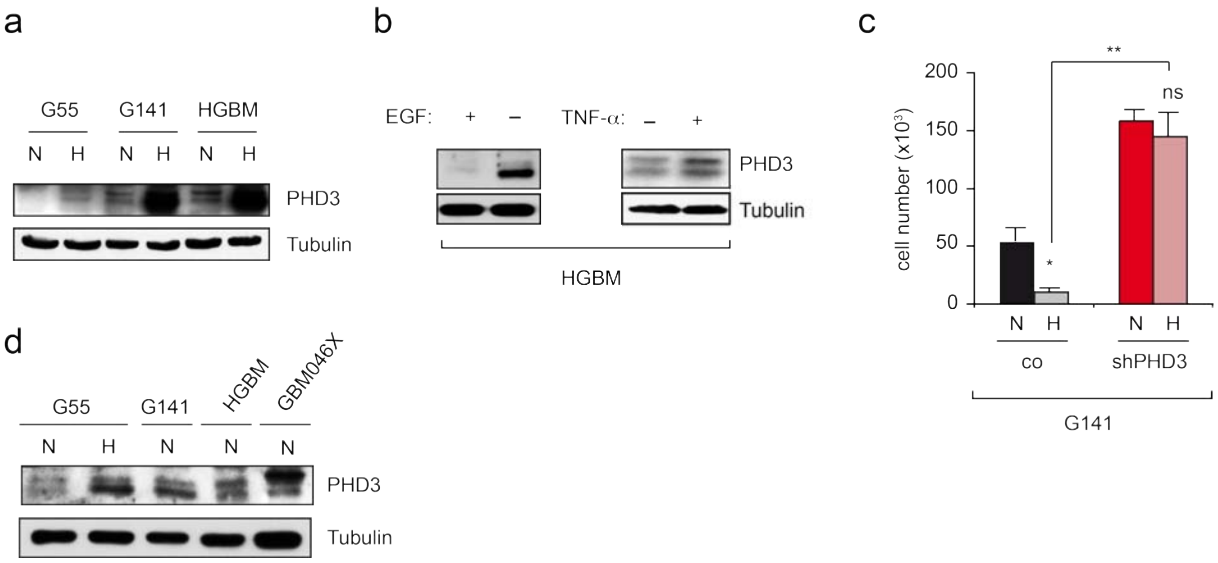

Supplementary Figure 7 │ PHD3 is a mediator of growth inhibitory signals

a, Different levels of hypoxic PHD3 induction in glioblastoma cell lines. Immunoblot of G55, G141 and HGBM tumour cells following exposure to 21% (N) or 1% O_2 (H) for 6 h. **b,** Growth inhibitory signals induce PHD3 expression. Immunoblot of HGBM tumour cells ± incubation with EGF (20 ng/ml) or TNFα (10 ng/ml). **c,** PHD3 loss protects against hypoxic growth inhibition. Cell accumulation (n=3) of G141 tumour cells expressing control or PHD3 shRNA was quantified following exposure to 21% (N) or 1% O_2 (H) for 3 days. **d,** Levels of endogenous PHD3 in G55, G141, HGBM glioma lines and the primary glioblastoma line GBM046x. Western Blots images (**a, b, d**) have been cropped for presentation. Full size images are presented in Supplementary Fig. 12. All values are means + SEM, * $p<0.05$; ** $p<0.01$.

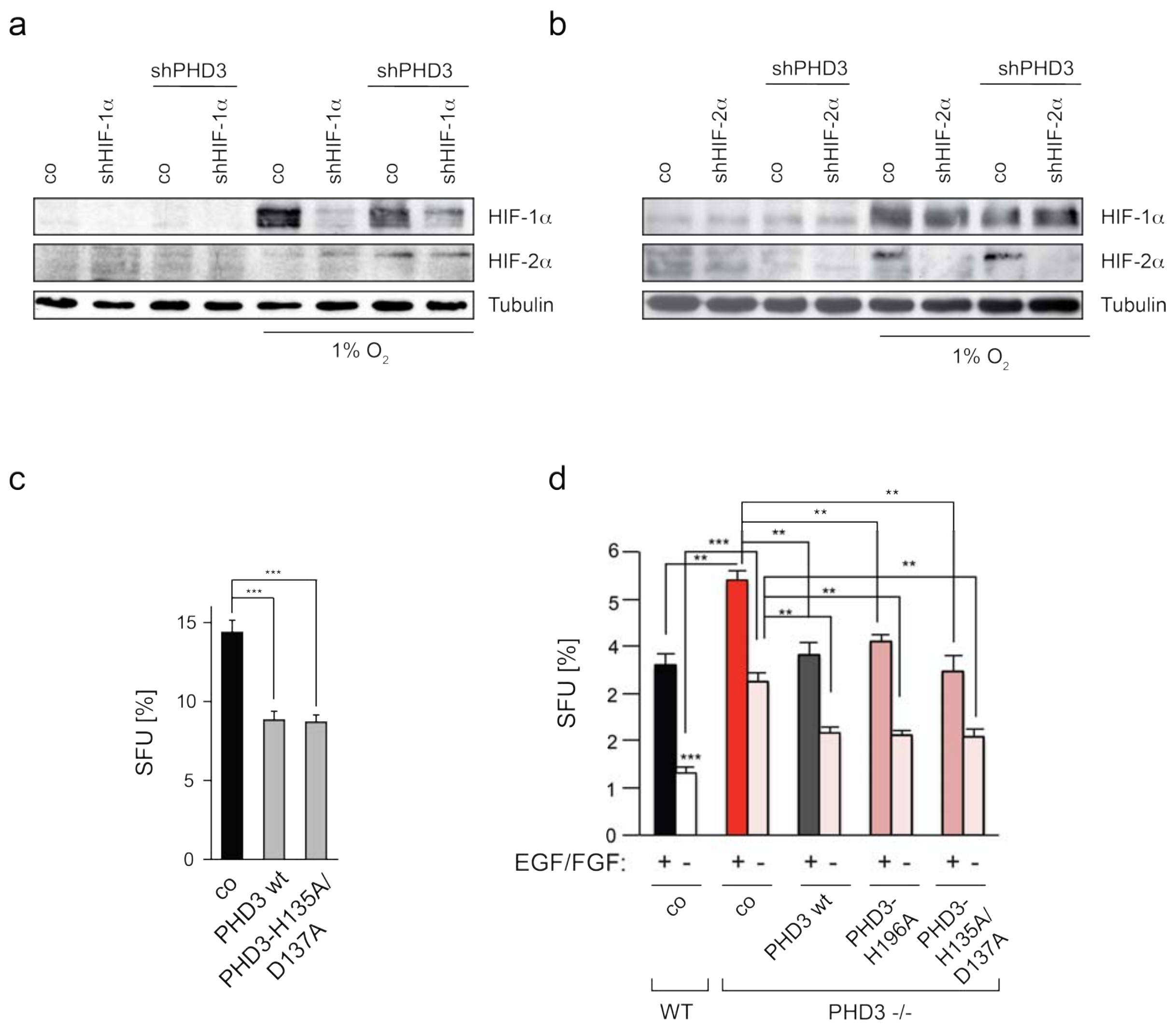

Supplementary Figure 8 │ Growth inhibition by PHD3 is hydroxylase/HIF independent

a, b, Immunoblot of G55 cells expressing PHD3 (or control) shRNA in combination with control, HIF-1α (**a**) or HIF-2α (**b**) shRNA following exposure to 21% or 1% O_2 for 24 h. **c,** G55 cells were transfected with either wild-type PHD3, the hydroxylase mutant PHD3-H135A/D137A or empty vector control, cultured as spheroids in B27 supplemented serum-free medium ± EGF/FGF and the number of spheroids was quantified after 4 days (n=6). **d,** PHD3-/- astrocytomas were transfected with either wild-type PHD3, the hydroxylase mutants PHD3-H196A or PHD3-H135A/D137A or empty vector control, cultured as spheroids in B27 supplemented serum-free medium ± EGF/FGF and the number of spheroids was quantified

after 4 days (n=6). Both WT and hydroxylase mutants revert the growth advantage of PHD3 loss in the sphere formation assay. Western Blots images (**a, b**) have been cropped for presentation. Full size images are presented in Supplementary Fig. 12. All values are means + SEM, ** $p<0.01$; ***$p<0.001$.

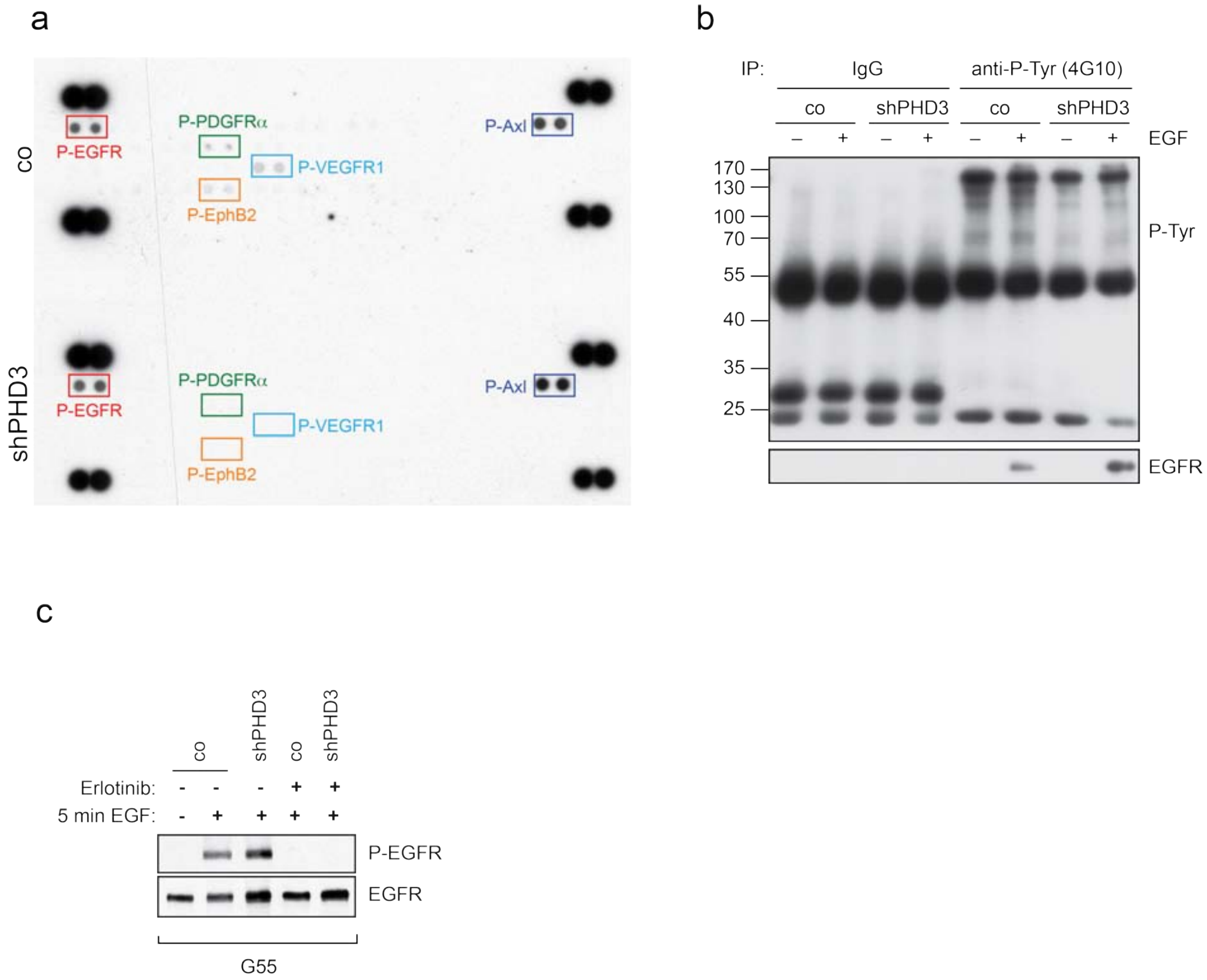

Supplementary Figure 9 │ PHD3 loss results in EGFR hyperphosphorylation

a, Silencing of PHD3 specifically promotes EGFR tyrosine phosphorylation. Extracts from non-stimulated G55 cells expressing control or PHD3 shRNA were analyzed with a human phospho-RTK array. PHD3 silencing induces an increase in EGFR phosphorylation, but the phosphorylation of other RTKs, such as PDGFRα, VEGFR1 or EphB2 was reduced. **b,** Silencing of PHD3 does not lead to a general increase in tyrosine phosphorylation. Extracts from G55 cells expressing control or PHD3 shRNA that were non-stimulated or stimulated for 5 min with 20 ng/ml EGF were immunoprecipitated with an anti-phosho-tyrosine antibody or IgG control. The immunoprecipitates were then probed with an anti-phospho-tyrosine antibody (top) or with an anti-EGFR antibody (bottom), revealing that while the total phospho-tyrosine signal is reduced by PHD3 silencing, EGFR phosphorylation is increased. **c,**

Erlotinib inhibits EGFR activation. Immunoblot of G55 cells expressing control or PHD3 shRNA ± stimulation with EGF for 5 min after 48 h of starvation. Where indicated, cells were pretreated with erlotinib (20 μM) for 2 h. Western Blots images (**b, c**) have been cropped for presentation. Full size images are presented in Supplementary Fig. 12.

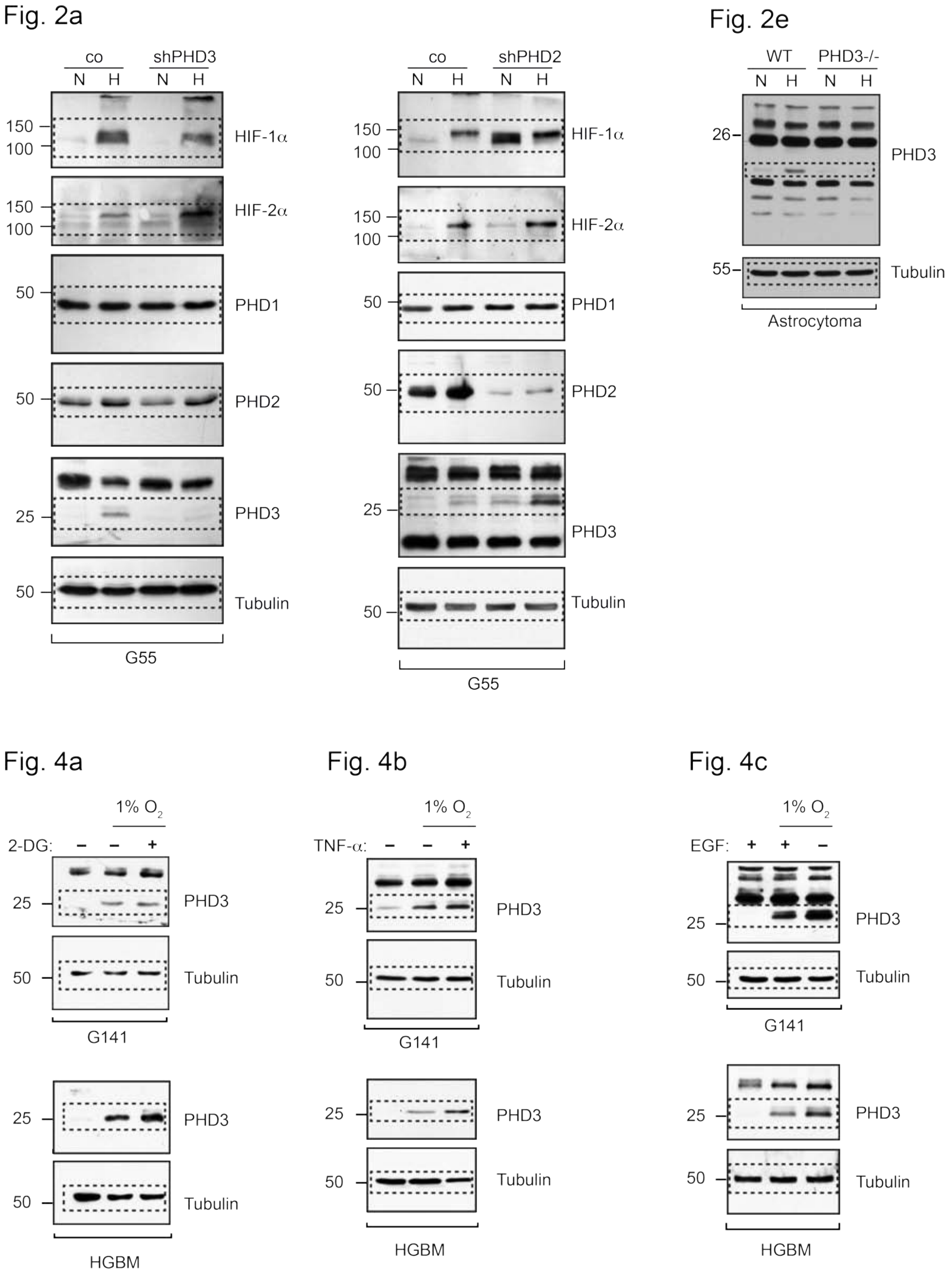

Supplementary Figure 10│ Uncropped, full-size scans of Western Blots

Uncropped, full-size scans of Western Blots shown in Fig. 2a, e and Fig. 4a-c.

Fig. 6a

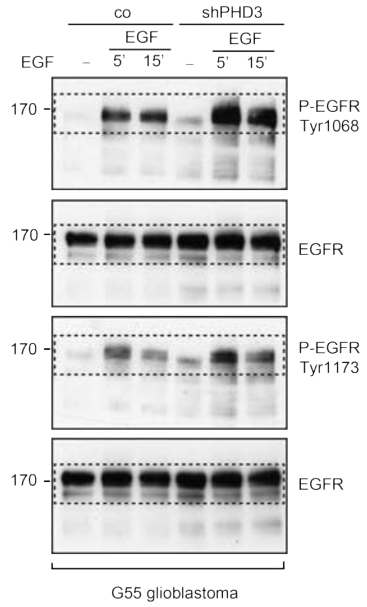

Suppl. Fig. 2b

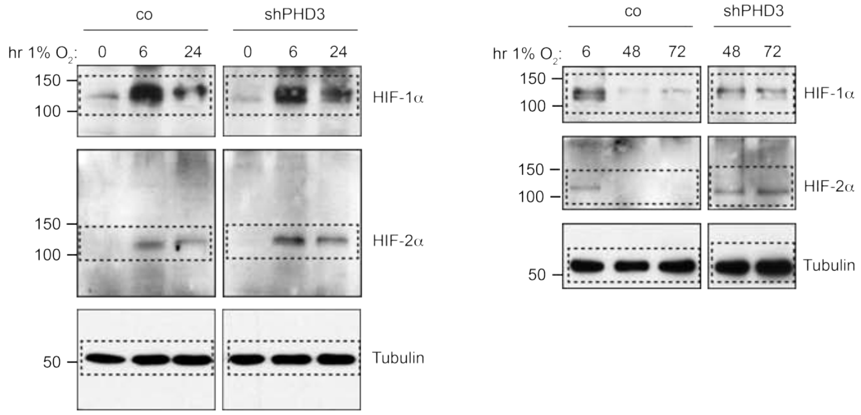

Suppl. Fig. 3a

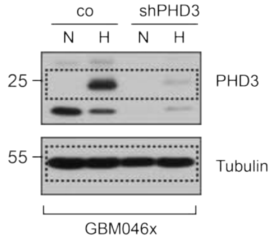

Suppl. Fig. 6b

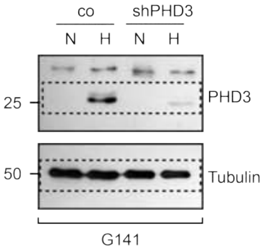

Suppl. Fig. 6c

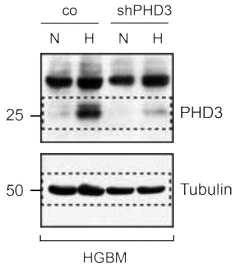

Supplementary Figure 11 │ Uncropped, full-size scans of Western Blots

Uncropped, full-size scans of Western Blots shown in Fig. 6a, Supplementary Fig. 2b, Supplementary Fig. 3a and Supplementary Fig. 6b, c.

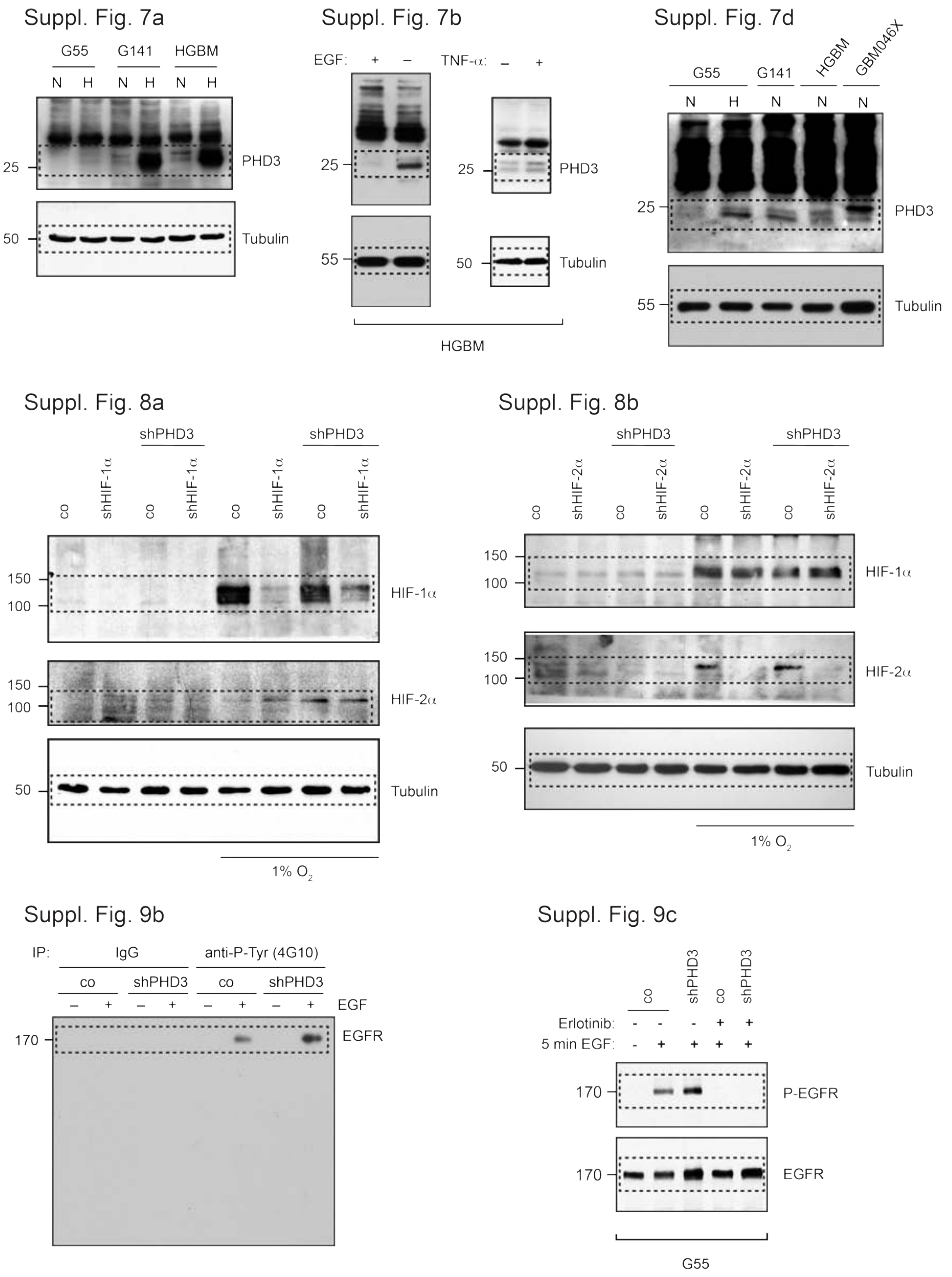

Supplementary Figure 12 │ Uncropped, full-size scans of Western Blots

Uncropped, full-size scans of Western Blots shown in Supplementary Fig. 7a, b, d, Supplementary Fig. 8a, b and Supplementary Fig. 9 b, c.

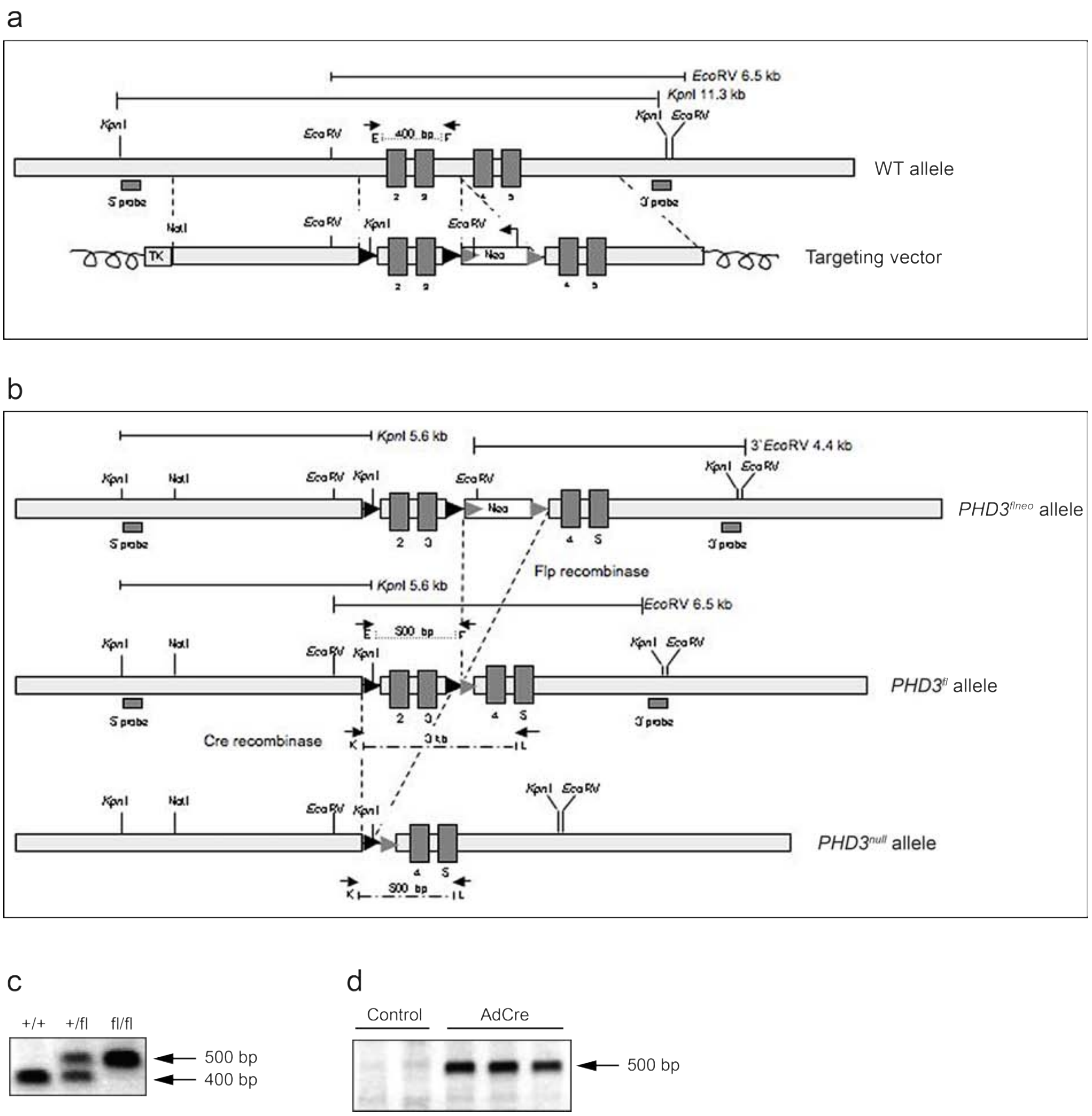

Supplementary Figure 13 | Generation of PHD3fl/fl mice and PHD3 excision

a, b, Schematic of the targeting strategy to generate *Phd3* conditional knockout mice, depicting the different *Phd3* alleles and the genotyping strategy. **a,** Top: Wild type *Phd3* allele, indicating the relative position of exon 2 to 5 (dark boxes in the genomic structure). Bottom: Outline of the *Phd3*^{flneo} targeting vector including a thymidine kinase cassette outside of the homology flanks, a 3.5 kb 5' homology flank, a loxP (dark arrowheads) flanked 2.6 kb fragment spanning exon 2 and 3, an FRT (grey arrowheads) flanked neo^{r} cassette, followed by a 3-kb 3' homology arm. The neo^{r} cassette was cloned in reverse orientation compared to

that of the *Phd3* gene. **b,** Top: *Phd3^{flneo}* allele after homologous recombination. Middle: *Phd3^{fl}* allele after Flp recombinase-mediated excision of the neo^{r} cassette. Bottom: *Phd3^{null}* allele after Cre recombinase-mediated excision of the fragment containing exon 2 and 3. Primers E and F (arrows) are used for PCR genotyping of the colony, discriminating between wild type and *Phd3^{fl}* alleles (size indicated by the dotted line above the alleles, see also **a, c**). PCR genotyping with primers K and L (arrows) was applied to distinguish between the *Phd3^{fl}* and *Phd3^{null}* alleles (size indicated by the discontinuous line below the alleles; see also **d**). **c,** Identification of floxed *Phd3* mice by PCR on genomic DNA from tail biopsies. PCR products amplified with primers E and F (see **a**, **b**) generating a 500 bp and 400 bp amplicon from *Phd3^{fl}* and WT alleles, respectively. **d,** PCR genotyping of *Phd3^{fl}* and *Phd3^{null}* mice after systemic adenoviral Cre recombinase (AdCre) injection. PCR products were obtained using genomic DNA prepared from liver. The excision of the fragment containing exons 2 and 3 in *Phd3^{fl}* mice results in the appearance of a 500-bp band (PCR with primers K and L, see **b**). The results of 2 or 3 representative mice per genotype are shown, reflecting the reproducibility and efficiency of Cre-mediated excision in vivo.

Supplementary Methods

Primer and shRNA sequences

a) Methylation Specific PCR:

Primer name	Sequence (5'-3')
PHD3-MF1	GGTTTTGGGAAGTCGATATATAAC
PHD3-MR1	CGACAAACTAATCCTCTACTACGAA
PHD3-UF1	GGTTTTGGGAAGTTGATATATAATG
PHD3-UR1	CAACAAACTAATCCTCTACTACAAA

b) miRNA (shRNA) sequences:

miPHD3 703 top sequence:

TGCTGTAACCTGTTCCATTTCCCGGAGTTTTGGCCACTGACTGACTCCGGGAAGG

AACAGGTTA

miPHD3 703 bottom sequence:

CCTGTAACCTGTTCCTTCCCGGAGTCAGTCAGTGGCCAAAACTCCGGGAAATGGA

ACAGGTTAC

miPHD2 1020 top sequence:

TGCTGTCAACATGACGTACATAACCCGTTTTGGCCACTGACTGACGGGTTATGCG

TCATGTTGA

miPHD2 1020 bottom sequence:

CCTGTCAACATGACGCATAACCCGTCAGTCAGTGGCCAAAACGGGTTATGTACGT

CATGTTGAC

SIMA top sequence:

TGCTGCATGAATATCTCTGTCTCCTTGTTTTGGCCACTGACTGACAAGGAGACAG

ATATTCATG

SIMA bottom sequence:

CCTGCATGAATATCTGTCTCCTTGTCAGTCAGTGGCCAAAACAAGGAGACAGAGA

TATTCATGC

c) shRNA sequences:

shHIF1α:

TGCTGTTGACAGTGAGCGCCGACACAGCCTGGATATGAAATAGTGAAGCCACAG

ATGTATTTCATATCCAGGCTGTGTCGATGCCTACTGCCTCGGA

shHIF2α:

TGCTGTTGACAGTGAGCGACCACCTTCAATGACTTCTAATTAGTGAAGCCACAGA

TGTAATTAGAAGTCATTGAAGGTGGGTGCCTACTGCCTCGGA

Supplementary References:

1. Toedt G, *et al.* Molecular signatures classify astrocytic gliomas by IDH1 mutation status. *Int J Cancer* **128**, 1095-1103 (2011).
